# Enhanced Capacitive Deionization of Hollow Mesoporous Carbon Spheres/MOFs Derived Nanocomposites by Interface‐Coating and Space‐Encapsulating Design

**DOI:** 10.1002/advs.202403802

**Published:** 2024-08-14

**Authors:** Yijian Tang, Yuxin Shi, Yichun Su, Shuai Cao, Jinliang Hu, Huijie Zhou, Yangyang Sun, Zheng Liu, Songtao Zhang, Huaiguo Xue, Huan Pang

**Affiliations:** ^1^ School of Chemistry and Chemical Engineering Yangzhou University Yangzhou Jiangsu 225009 P. R. China; ^2^ Jiangsu Yangnong Chemical Group Co. Ltd. Yangzhou 225009 P. R. China

**Keywords:** capacitive deionization, hollow mesoporous carbon spheres, MOF‐derived carbons, template method

## Abstract

Exploring new carbon‐based electrode materials is quite necessary for enhancing capacitive deionization (CDI). Here, hollow mesoporous carbon spheres (HMCSs)/metal‐organic frameworks (MOFs) derived carbon materials (NC(M)/HMCSs and NC(M)@HMCSs) are successfully prepared by interface‐coating and space‐encapsulating design, respectively. The obtained NC(M)/HMCSs and NC(M)@HMCSs possess a hierarchical hollow nanoarchitecture with abundant nitrogen doping, high specific surface area, and abundant meso‐/microporous pores. These merits are conducive to rapid ion diffusion and charge transfer during the adsorption process. Compared to NC(M)/HMCSs, NC(M)@HMCSs exhibit superior electrochemical performance due to their better utilization of the internal space of hollow carbon, forming an interconnected 3D framework. In addition, the introduction of Ni ions is more conducive to the synergistic effect between ZIF(M)‐derived carbon and N‐doped carbon shell compared with other ions (Mn, Co, Cu ions). The resultant Ni‐1‐800‐based CDI device exhibits excellent salt adsorption capacity (SAC, 37.82 mg g^−1^) and good recyclability. This will provide a new direction for the MOF nanoparticle‐driven assembly strategy and the application of hierarchical hollow carbon nanoarchitecture to CDI.

## Graphical abstract

Two different types of zeolite imidazole frameworks (ZIF)(M)‐derived nitrogen‐doped carbon and hollow mesoporous carbon nanospheres (HMCSs) composites (nitrogen‐doped carbons (NC)(M)/HMCSs, NC(M)@HMCSs) are synthesized through interface‐coating and space‐encapsulating design, respectively, and their electrochemical performance are explored. In addition, the influence of Ni ions doping on capacitive deionization (CDI) performance for NC(Zn, Ni)@HMCSs is explored.

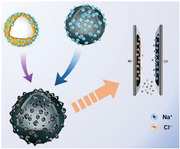

## 1 Introduction

Hollow mesoporous carbon nanospheres (HMCSs) possess advantageous properties such as low density, porous shells, accessible interior space, high surface area, and large pore volume,^[^ ^1^, ^2^, ^3^ ^]^ which make them a member of the multi‐functional advanced materials. Due to their unique physical and chemical properties, HMCSs have certain potential in applications such as catalysis,^[^ ^4^, ^5^, ^6^ ^]^ adsorption,^[^ ^7^, ^8^, ^9^ ^]^ and energy storage and conversion.^[^ ^10^, ^11^, ^12^ ^]^ The key to the successful application of HMCSs in various directions is based on precise control of shell thickness, cavity inner diameter, surface chemical properties, and stability in the medium.^[^ ^13^, ^14^, ^15^ ^]^ In general, there are various strategic synthesis methods for the preparation of hollow carbon nanospheres, such as hard‐templating approach,^[^ ^16^ ^]^ soft‐templating approach,^[^ ^17^ ^]^ self‐assembly method,^[^ ^18^ ^]^ and Stöber‐based process.^[^ ^19^ ^]^ Previously, most of the research focus had been on the synthesis of hierarchical hollow carbon nanoarchitecture with tunable sizes. Nevertheless, little attention has been paid to the surface modification and internal cavities of HMCSs.

The modification of HMCSs‐based nanomaterials can offer some advantages and potential applications. First, the presence of abundant meso‐/microporous pores on the surface of carbon shells can provide a pathway for mass diffusion, thereby reducing diffusion resistance.^[^ ^20^, ^21^, ^22^ ^]^ Second, the excellent stability and durability of the carbon framework enable them to withstand harsh reaction conditions, further enhancing their surface modifiability.^[^ ^23^, ^24^ ^]^ Lastly, HMCSs, as ideal nanoreactors with internal cavities, provide not only a huge reaction site, but also a buffer space for the volume expansion of sustainably active materials.^[^ ^25^, ^26^, ^27^ ^]^ Surface modification and spatial confinement are two commonly methods to modify hollow materials. The interface‐coating method usually include nanoparticle self‐assembly and molecule modification, which will further affect the phase structure, chemical composition and shell microstructure of modified HMCSs‐based nanomaterials.^[^ ^28^ ^]^ The space‐encapsulating method usually confines the guest substances within the internal space of the host material (HMCSs), fully utilizing the internal space of HMCSs to better form a heterostructure.^[^ ^29^ ^]^ Therefore, the study of surface modification and internal space of HMCSs‐based nanomaterials is of certain significance.

Zeolite imidazole frameworks (ZIFs), as a subclass of MOFs, are assembled from transition metals and imidazole ligands.^[^ ^30^ ^]^ In recent years, ZIFs have been considered as ideal precursors for synthesizing porous carbon materials through calcination,^[^ ^31^, ^32^, ^33^ ^]^ which are applied in the fields such as CDI,^[^ ^34^, ^35^, ^36^ ^]^ supercapacitors,^[^ ^37^, ^38^ ^]^ batteries,^[^ ^39^, ^40^ ^]^ and so on. The particle size, complex architectures, and chemical composition of ZIFs can be easily adjusted to produce different carbon materials. In addition, the nitrogen atoms in imidazole ligands can be well retained and doped into the synthesized carbon materials.^[^ ^41^ ^]^ Furthermore, the introduction of different metal ions can promote changes in the chemical composition and graphitization degree of ZIFs‐derived carbons, further affecting them electron/ion transport and accessible ion adsorption sites.^[^ ^42^, ^43^, ^44^, ^45^ ^]^ For example, Co/Zn‐ZIFs^[^ ^46^, ^47^ ^]^ as precursors are common bimetallic MOFs used in CDI due to the similar tetrahedral coordination mechanism between the two metals. In addition, the incorporation of Fe or Ni metals into Co‐ZIFs^[^ ^48^ ^]^ can improve the conductivity of derived carbons, which is beneficial for CDI performance. In all, reasonable design of ZIFs‐derived carbons and HMCSs composites is beneficial for improving their capacitance performance and electro‐sorption capacity.

Herein, HMCSs/MOFs‐derived carbon materials have been successfully prepared by interface‐coating and space‐encapsulating design. The interface‐coating design is as follows: ZIFs nanocrystals were coated onto silica‐polydopamine (SiO_2_@SiO_2_+PDA) spheres and further carbonized and etched to obtain a novel porous carbon material (denoted NC(M)/HMCSs), in which nitrogen‐doped carbons (denoted NC(M)) were derived from a zeolitic imidazolate framework (denoted ZIF(M)) grown onto HMCSs. In addition, the design route for space‐encapsulating is to first carbonize and etch SiO_2_@SiO_2_+PDA spheres to obtain HMCSs, then limit the growth of ZIF(M) within the HMCSs to obtain ZIF(M)@HMCSs, and finally further carbonize them to obtain another novel porous carbon material (denoted NC(M)@HMCSs). Furthermore, a series of HMCSs/ZIFs‐derived carbon materials were prepared by introducing different metal sources and adjusting the proportion of metal ions. During the pyrolysis process, most of the Zn species are eliminated, while trace doped metals (Mn, Co, Ni, Cu) loaded on HMCSs and NC(M). The existence of Ni metal is more conducive to the synergistic effect between ZIF(M)‐derived carbon and N‐doped carbon shell compared with other metals. The electrochemical results indicate that NC(Zn, Ni)@HMCSs with hollow core‐shell structure exhibits better electrochemical performance compared with others. The introduction of Ni^2+^ can greatly accelerate the degree of graphitization of the carbon layer, thereby accelerating the electron mobility of composites. These carbonaceous composites with slight Ni doping were further used as electrode materials for CDI units. The resultant Ni‐1‐800 (Ni/Zn = 1/10) based CDI device exhibits excellent SAC of 37.82 mg g^−1^ and high maximum desalination rate of 7.73 mg g^−1^ min^−1^. This work will provide a direction for the design of MOFs‐derived carbon/hollow carbon‐based composites in the CDI application.

## 2 Results and Discussion

### 2.1 Characteristics

**Figure 1:**
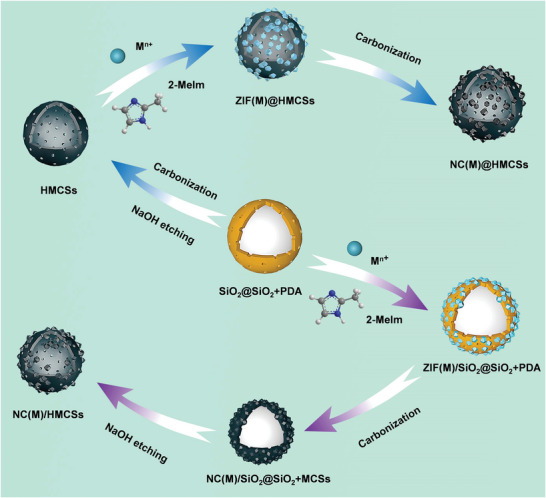
Schematic illustration of the synthesis processes of NC(M)/HMCSs and NC(M)@HMCSs.

The synthetic methods for the NC(M)/HMCSs and NC(M)@HMCSs materials are illustrated in **Figure** **1**. First, tetraethyl orthosilicate underwent hydrolysis and condensation to form silica primary particles. Second, silica primary particles and polydopamine (PDA) were cocondensed onto SiO_2_ spheres to obtain the SiO_2_@SiO_2_+PDA spheres (Figure S1, Supporting Information). Next, NC(M)/HMCSs and NC(M)@HMCSs nanocomposites were prepared through two design routes (interface‐coating and space‐encapsulating design).

**Figure 2:**
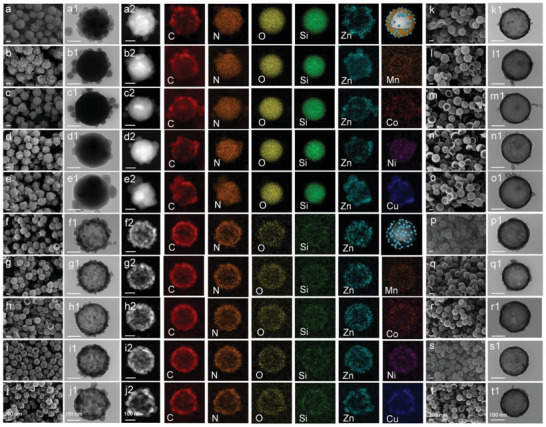
SEM (first column), TEM (second column) and the corresponding EDS element mapping images of a,a1,a2) ZIF(Zn)/SiO_2_@SiO_2_+PDA, b,b1,b2) ZIF(Zn, Mn)/SiO_2_@SiO_2_+PDA, c,c1,c2) ZIF(Zn, Co)/SiO_2_@SiO_2_+PDA, d,d1,d2) ZIF(Zn, Ni)/SiO_2_@SiO_2_+PDA, e,e1,e2) ZIF(Zn, Cu)/SiO_2_@SiO_2_+PDA, f,f1,f2) ZIF(Zn)@HMCSs, g,g1,g2) ZIF(Zn, Mn)@HMCSs, h,h1,h2) ZIF(Zn, Co)@HMCSs, i,i1,i2) ZIF(Zn, Ni)@HMCSs, and j,j1,j2) ZIF(Zn, Cu)@HMCSs. SEM (penultimate column), TEM (last column) images of k,k1) NC (Zn)/ HMCSs, l,l1) NC(Zn, Mn)/HMCSs, m,m1) NC(Zn, Co)/HMCSs, n,n1) NC (Zn, Ni)/HMCSs, o,o1) NC(Zn, Cu)/HMCSs, p,p1) NC(Zn)@HMCSs, q,q1) NC(Zn, Mn)@HMCSs, r,r1) NC(Zn, Co)@HMCSs, s,s1) NC(Zn, Ni)@HMCSs, and t,t1) NC(Zn, Cu)@HMCSs.

The interface‐coating design is as follows: ZIF(M) nanocrystals were in situ grown on the surface of SiO_2_@SiO_2_+PDA spheres to generate ZIF(M)/SiO_2_@SiO_2_+PDA. Here, zinc nitrate and other nitrates (manganese nitrate, cobalt nitrate, nickel nitrate, or copper nitrate) were thoroughly mixed with SiO_2_@SiO_2_+PDA spheres, ensuring that zinc ions and other metal ions (Mn^2+^, Co^2+^, Ni^2+^, or Cu^2+^) could be well attached to the surface of nanosphere and coordinated with 2‐methylimidazole (2‐MeIm). The structure and morphology of ZIF(M)/SiO_2_@SiO_2_+PDA were observed by using scanning electron microscopy (SEM) and transmission electron microscopy (TEM). Small‐sized ZIF(M) nanocrystals (ZIF(Zn), ZIF(Zn, Mn), ZIF(Zn, Co), ZIF(Zn, Ni), and ZIF(Zn, Cu)) were dispersedly grown onto SiO_2_@SiO_2_+PDA spheres to form a ZIF(M)/SiO_2_@SiO_2_+PDA (**Figure** **2**a–e) heterostructure. TEM images (Figure 2a1–e1) illustrate that ZIF(M) nanoparticles with an average diameter of about 30 nm are coated onto the nanospheres (≈280 nm), and there are many nano spaces between ZIF(M) nanoparticles. The energy dispersive spectrometry (EDS) elemental mapping images (Figure 2a2–e2) of ZIF(M)/SiO_2_@SiO_2_+PDA show the existence of C, N, O, Si, Zn, and the corresponding doped metal elements (Mn, Co, Ni, and Cu), which clearly show that doped metal ions are distributed on the surface of SiO_2_@SiO_2_+PDA spheres. The crystal structure of the ZIF(M)/SiO_2_@SiO_2_+PDA was analyzed by X‐ray diffraction (XRD, Figures S2 and S3, Supporting Information). The corresponding characteristic peaks are generally consistent with those of pure ZIF‐8 (ZIF(Zn)) nanocrystals,^[^ ^49^, ^50^ ^]^ indicating that ZIF(M) nanocrystals were successfully formed on the nanospheres. Next, ZIF(M)/SiO_2_@SiO_2_+PDA nanospheres were converted to SiO_2_@carbon nanospheres (NC(M)/SiO_2_@SiO_2_+MCSs, Figure S4, Supporting Information) by a carbonization method. The nano spaces between ZIF(M) nanoparticles would be converted to mesopores. Eventually, NC(M)/SiO_2_@SiO_2_+MCSs were completely etched in NaOH solution to remove the silica template to obtain NC(M)/HMCSs (Figure 2k–o). TEM images (Figure 2k1–o1) illustrate that ZIF(M)‐derived nitrogen‐doped carbons (NC(M)) coat onto thin carbon shells (≈10 nm) and the cavity is approximately 250 nm. In addition, pure ZIF‐8 (Figure S5, Supporting Information) nanocrystals were obtained by coordinating zinc nitrate with 2‐MeIm. The ZIF‐8 nanoparticles were calcined to obtain NC(Zn) with slight surface collapse (Figure S6, Supporting Information), resulting in a slight decrease in particle size. Another space‐encapsulating design is as follows: SiO_2_@SiO_2_+PDA spheres were successively calcined and etched to obtain HMCSs. As shown in Figure S7 Supporting Information), the cavity of the HMCSs is about 250 nm, providing a large space for the growth of ZIF(M). Then, zinc nitrate and other nitrates were thoroughly mixed with HMCSs by ultrasonic and stirring methods. Next, add 2‐MeIm to coordinate with metal ions to form ZIF(M)@HMCSs (Figure 2f–j). TEM images (Figure 2f1–j1) show that ZIF(M) nanoparticles (≈10 nm) grow well within HMCSs. The XRD patterns (Figure S8, Supporting Information) of ZIF(M)@HMCSs show that all diffraction peaks can be attributed to ZIF(M). There is no significant deviation in the positions of these characteristic peaks with the doping of metal ions. EDS elemental mapping images (Figure 2f2–j2) of ZIF(M)@HMCSs show the existence of C, N, O, Si, Zn, and the corresponding doped metal elements, which clearly show that the Si element is hardly existent due to the etching technique and the Zn element content is significantly higher than other trace doped metal elements. Finally, ZIF(M)@HMCSs were converted to NC(M)@HMCSs (Figure 2p–t) by a carbonization method. As shown in Figure 2p1–t1, various types of NC(M) are well confined into HMCSs, forming an interconnected 3D framework.

**Figure 3:**
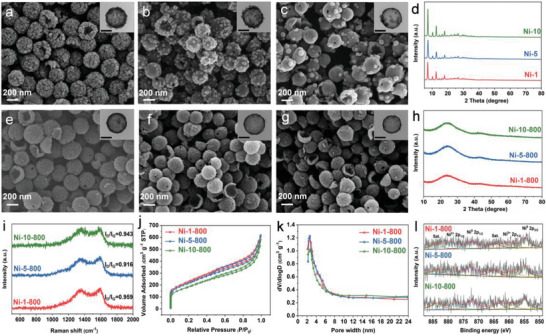
SEM images of a) Ni‐1, b) Ni‐5, and c) Ni‐10, (inset) TEM images of the corresponding samples (scale bar 100 nm). d) XRD patterns of Ni‐1, Ni‐5, and Ni‐10 samples. SEM images of e) Ni‐1‐800, f) Ni‐5‐800, and g) Ni‐10‐800, (Inset) TEM images of the corresponding samples (scale bar 100 nm). h) XRD patterns, i) Raman spectrums, j) N_2_ adsorption–desorption isotherms, k) BJH pore size distribution, and l) High‐resolution Ni 2p spectrums of Ni‐1‐800, Ni‐5‐800, and Ni‐10‐800 samples.

In order to explore the effect of the metal ion doping content on the morphology of ZIF(M)@HMCSs, the morphology was observed by changing the nickel nitrate content. As shown in **Figure** **3**a–c, with the increase content of Ni^2+^, the size of ZIF(Zn, Ni) nanoparticles grown in HMCSs increases, while the number of particles also decreases. This may lead to an increase in the nano spaces between ZIF(Zn, Ni) nanoparticles, which in turn leads to the inability of the nanospace to be converted into mesopores after carbonization. The XRD patterns (Figure 3d) of these samples show that all characteristic peaks can be attributed to ZIF‐8. The positions of these characteristic peaks have no change with the amount of Ni^2+^ doping content. No characteristic peaks corresponding to amorphous carbon were found, which may be covered by the sharp characteristic peaks of ZIF‐8. Further carbonization of ZIF(Zn, Ni)@HMCSs resulted in a series of NC(Zn, Ni)@HMCSs materials (Figure 3e–g), which also exhibited the aforementioned NC(M)@HMCSs morphology characteristics. All NC(M)@HMCSs samples (Figure S9, Supporting Information and Figure 3h) exhibit two broad peaks at approximately 24° (002) and 43° (100), with no other obvious crystal phases. The two broad peaks can be attributed to the amorphous carbon frameworks,^[^ ^51^ ^]^ and the absence of peaks for metallic phases is due to the basic evaporation of zinc during the calcination process and the trace doping of other metal ions. Figure 3i and Figure S10 (Supporting Information) exhibit Raman spectrums of the NC(M)@HMCSs. Two broad peaks at approximately 1350 and 1590 cm^−1^ can correspond to D‐ and G‐band, respectively.^[^ ^52^ ^]^ The intensity ratio of D‐band to G‐band (*I*_D_/*I*_G_) is often used to measure the degree of carbon disorder and defects.^[^ ^53^ ^]^ Obviously, *I*_D_/*I*_G_ for NC(Zn)@HMCSs, NC(Zn, Mn)@HMCSs, NC(Zn, Co)@HMCSs, NC(Zn, Ni)@HMCSs, and NC(Zn, Cu)@HMCSs are 0.956, 0.939, 0.947, 0.959, and 0.922, respectively, indicating that the doping of other metal ions leads to the reduction of defects in NC(Zn)@HMCSs. Comparatively, the doping of trace Ni^2+^ has little effect on the degree of carbon disorder. However, the increase in Ni^2+^ ions doping is not conducive to the enhancement of carbon disorder of NC(Zn, Ni)@HMCSs (Ni‐1‐800: 0.959, Ni‐5‐800: 0.916, and Ni‐10‐800: 0.943). Therefore, Ni‐1‐800 sample possesses more defects and may promote charge accumulation, thereby facilitating charge transfer during the adsorption process.

The specific surface area (SSA) and pore structures of the hybrid materials were explored by N_2_ adsorption–desorption isotherms (Table S1, Supporting Information). As shown in Figures S11 and S12 (Supporting Information), the SSA of ZIF(M)/SiO_2_@SiO_2_+PDA is basically larger than that of SiO_2_@SiO_2_+PDA, which indicates that the existence of ZIF(M) can improve the pore structure of SiO_2_@SiO_2_+PDA to a certain extent. All ZIF(M)@HMCSs materials (Figure S14, Supporting Information) exhibit adsorption characteristics of hierarchical pore nanoarchitecture. The hysteresis loops of ZIF(M)@HMCSs decreases compared to HMCSs, proving that ZIF(M) nanoparticles occupy the cavity of HMCSs. The N_2_ adsorption‐desorption curves of ZIF(M)@HMCSs show type IV isotherm. Combined with the pore size distribution (Figure S15, Supporting Information), ZIF(M)@HMCSs possess abundant mesopores, mainly distributed in 2–8 nm. Obviously, compared with ZIF(M)@HMCSs precursor, NC(M)@HMCSs possess lower SSA, and larger pore size. The possible reason is that high‐temperature pyrolysis leads to the collapse of the ZIF(M) structure, and Zn evaporation causes some small mesopores to become larger mesopores. The SSA and pore volumes of NC(Zn)/HMCSs (1388.41 m^2^ g^−1^ and 1.38 cm^3^ g^−1^) and NC(Zn)@HMCSs (844.27 m^2^ g^−1^ and 0.96 cm^3^ g^−1^) are much larger than those of NC(Zn) (557.36 m^2^ g^−1^ and 0.52 cm^3^ g^−1^, Figure S13, Supporting Information), which is mainly due to the co‐presence of N‐doped hollow mesoporous carbon spheres. In addition, the SSA of Ni‐1‐800 with the least Ni content is larger than that of Ni‐5‐800 and Ni‐10‐800 (Figure 3j). The N_2_ adsorption–desorption curves of those NC(Zn, Ni)@HMCSs also show type IV structure, further confirming their mesoporous structure. As shown in Figure 3k, those NC(Zn, Ni)@HMCSs samples exhibit abundant mesopores and the pore size centered at about 2–6 nm. The high SSA and such hierarchical pore nanoarchitecture are significant to facilitate the accessibility of adsorption sites and ion transport, which is conducive to the CDI process.^[^ ^54^ ^]^

The chemical compositions and valence states of the synthesized Ni‐*x*‐800 were analyzed by X‐ray photoelectron spectroscopy (XPS). The spectra shown in Figure S16 (Supporting Information) indicates the existence of C, N, O, Ni, and Zn in all hybrid materials. The high‐resolution XPS C 1 s spectra (Figure S17, Supporting Information) shows four peaks^[^ ^55^ ^]^ (C─C, C─N, C─O, and O─C═O bonds) corresponding to 284.8, 285.8, 287.5, and 289.1 eV, respectively. N1s spectra (Figure S18, Supporting Information) is deconvoluted into four peaks at about 398.6 eV (pyridinic‐N), 399.4 eV (pyrrolic‐N), 401.0 eV (graphitic‐N), and 402.4 eV (oxidized‐N). Figure 3l shows the XPS Ni 2p spectrum consisting of four typical peaks centered at approximately 872.6 (Ni^0^ 2p_1/2_), 854.7 eV (Ni^0^ 2p_3/2_), 879.9 (Ni^2+^ 2p_1/2_), 859.4 (Ni^2+^ 2p_3/2_) and two corresponding satellite peaks^[^ ^56^ ^]^ (883.6 and 864.9 eV), indicating the coexistence of Ni^0^ and Ni^2+^ in Ni‐1 800, Ni‐5 800, and Ni‐10 800 samples.^[^ ^57^, ^58^, ^59^ ^]^ From the XPS results, it can be confirmed that ZIF(Zn, Ni)‐derived NC(Zn, Ni) coexists with HMCSs.

### 2.2 Electrochemical Performance

**Figure 4:**
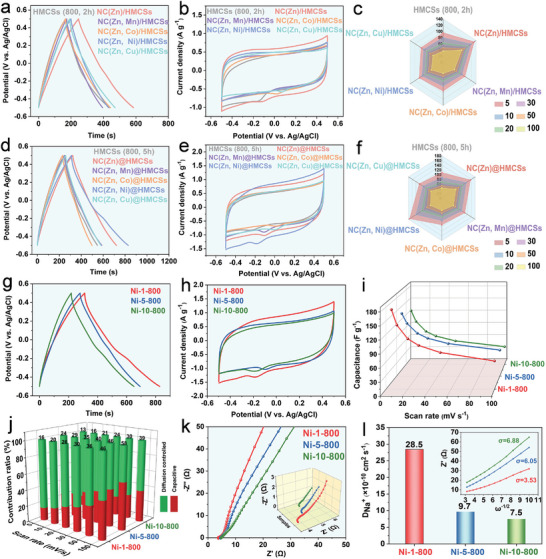
a) GCD diagrams at 0.5 A g^−1^, b) CV diagrams at 5 mV s^−1^, and c) the corresponding specific capacitances versus scan rates of HMCSs (800, 2 h), NC (Zn)/HMCSs, NC(Zn, Mn)/HMCSs, NC(Zn, Co)/HMCSs, NC(Zn, Ni)/HMCSs, and NC(Zn, Cu)/HMCSs in 1.0 m NaCl solution. d) GCD diagrams at 0.5 A g^−1^, e) CV diagrams at 5 mV s^−1^, and f) the corresponding specific capacitances versus scan rates of HMCSs (800, 5 h), NC(Zn)@HMCSs, NC(Zn, Mn)@HMCSs, NC(Zn, Co)@HMCSs, NC(Zn, Ni)@HMCSs, and NC(Zn, Cu)@HMCSs in 1.0 m NaCl solution. g) GCD diagrams at 0.5 A g^−1^, h) CV diagrams at 5 mV s^−1^, and i) The corresponding specific capacitances versus scan rates of Ni‐1‐800, Ni‐5‐800, Ni‐10‐800 in 1.0 m NaCl solution. j) Capacitive ratio of Ni‐1‐800, Ni‐5‐800, and Ni‐10‐800 electrode materials at various scan rates. k) EIS of the Ni‐1‐800, Ni‐5‐800, and Ni‐10‐800 electrodes. l) Na^+^ diffusion coefficients and the corresponding charts of *Z’*(Ω) versus w^−1⁄2^.

To evaluate the electrochemical performance of electrodes, a three‐electrode system was explored in 1.0 m NaCl solution. The galvanostatic charge‐discharge (GCD) curves of NC(Zn), HCMSs, NC(M)/HMCSs, and NC(M)@HMCSs samples shown in Figure S19a, S20, S22, S28 (Supporting information) display slightly distorted triangles, which can be attributed to the presence of pseudocapacitive behavior. **Figure** **4**a shows the GCD diagram of NC(M)/HMCSs at 0.5 A g^−1^, which clearly illustrates that the discharge time of NC(Zn)/HMCSs is longer than that of HMCSs (800, 2 h) and other NC(M)/HMCSs. The corresponding specific capacitance (F g^−1^) at 0.5 A g^−1^ are as follows: NC(Zn)/HMCSs (169.2) > NC(Zn, Cu)/HMCSs (140.6) > NC(Zn, Co)/HMCSs (138.7) > NC(Zn, Ni)/HMCSs (115.7) > NC(Zn, Mn)/HMCSs (111.2). This shows that the additional doping of other metal ions is not conducive to the improvement of capacitive properties of hybrid materials in the interface‐coating design. Figure 4a can also demonstrate that the capacitive performance of NC(Zn)/HMCSs (169.2 F g^−1^) hybrid materials is significantly better than that of HMCSs (800, 2 h) (135.9 F g^−1^) and NC(Zn) (147.3 F g^−1^), mainly due to the synergistic effect of N‐doped carbon shell and NC(Zn). Moreover, the GCD diagram of NC(M)@HMCSs at 0.5 A g^−1^ shown in Figure 4d illustrates that the specific capacitance performance (F g^−1^) of NC(Zn, Ni)@HMCSs (259.1) is superior to that of HMCSs (800, 5 h) (150.6) and other NC(M)@HMCSs. The corresponding specific capacitance (F g^−1^) at 0.5 A g^−1^ are as follows: NC(Zn, Ni)@HMCSs (259.1) > NC(Zn)@HMCSs (199.9) > NC(Zn, Mn)@HMCSs (169.2) > NC(Zn, Cu)@HMCSs (160.7) > NC(Zn, Co)@HMCSs (134.1). This indicates that the introduction of Ni ions is more conducive to the synergistic effect between ZIF(M)‐derived carbon and N‐doped carbon shell in the space‐encapsulating design.

The cyclic voltammetry (CV) curves (Figure 4b,e) of HCMSs, NC(M)/HMCSs, and NC(M)@HMCSs samples at 5 mV s^−1^ show irregular rectangular shapes, indicating pseudocapacitive behavior.^[^ ^60^ ^]^ Apparently, the CV curve of NC(Zn, Ni)@HMCSs shows the maximum integrated area, indicating the optimal capacitance characteristics, which is consistent with the above GCD curve analysis results. Figures S19b, S21, and S23 (Supporting Information) curves show CV plots of NC(Zn), HCMSs, NC(M)/HMCSs, and NC(M)@HMCSs at different scanning rates, from which the corresponding capacitance values can be calculated (Table S2, Supporting Information). Figure 4c,f shows that the specific capacitance decreases significantly as the scanning rate increases. The corresponding specific capacitance (F g^−1^) at 5 mV s^−1^ are as follows: NC(Zn, Ni)@HMCSs (174.5) > NC(Zn)@HMCSs (154.6) > NC(Zn)/HMCSs (131.5) > HMCSs (800, 5 h) (108.4) > NC(Zn, Ni)/HMCSs (101.4) > HMCSs (800, 2 h) (95.2). Based on the above, it can be concluded that the capacitive performance of NC(M)@HMCSs obtained through space‐encapsulating design is significantly better than that of NC(M)/HMCSs by interface‐coating design (the specific capacitance values shown in Tables S2 and S3, Supporting Information), which further demonstrates the significance of fully utilizing the internal cavities of HMCSs.

According to these above results, NC(Zn, Ni)@HMCSs obtained through space‐encapsulating design shows the optimal capacitance characteristics. The electrochemical properties of NC(Zn, Ni)@HMCSs electrodes with different nickel contents (Ni‐1‐800, Ni‐5‐800, and Ni‐10‐800) were further tested. As shown in Figure 4g, the discharge time of Ni‐1‐800 is longer than that of Ni‐5‐800 and Ni‐10‐800, indicating that trace doping of Ni ions is more conducive to capacitance characteristics. The doping of more nickel ions may cause certain damage to the carbonaceous structure, which is detrimental to the synergistic effect between ZIF(Zn, Ni)‐derived carbon and N‐doped carbon shells. Figure 4h shows the maximum CV curve integral area for Ni‐1‐800, indicating that this NC(Zn, Ni)@HMCSs possesses better specific capacitance. The specific capacitances (F g^−1^) at 5 mV s^−1^ are as follows: Ni‐1‐800 (174.5) > Ni‐5‐800 (144.3) > Ni‐10‐800 (129.5). The CV curves of Ni‐1‐800, Ni‐5‐800, and Ni‐10‐800 electrodes at different scan rates (Figure S24a–c, Supporting Information) retain the quasi‐rectangular shapes, proving that the hybrid materials display pseudocapacitive behaviors with excellent capacitive reversibility. The specific capacitance decreases with increasing scan rates (Figure 4i), and Ni‐1‐800 electrode shows the largest specific capacitance at different scan rates. In order to explore the electrochemical kinetic properties of NC(Zn, Ni)@HMCSs electrodes, diffusion control and capacitive behavior were analyzed in detail (Figures S25–S27, Supporting Information). Figure S24d (Supporting Information) shows that the *b* values of the Ni‐1‐800, Ni‐5‐800, and Ni‐10‐800 samples are 0.72, 0.79, and 0.74, respectively, indicating that the Ni‐1‐800 electrode is mainly affected by diffusion‐controlled behavior.^[^ ^61^ ^]^ As shown in Figure 4j, the capacitive contribution of Ni‐1‐800 electrode is increased from 16% to 46% with the increase of the scan rates. Notably, high specific capacitance can promote fast and reversible ion storage. The charge and ion transport kinetics of the Ni‐1‐800, Ni‐5‐800, and Ni‐10‐800 electrodes were also investigated by electrochemical impedance spectroscopy (EIS, Figure 4k,l). The Nyquist diagram in low‐frequency region shows that the Ni‐1‐800 electrode possesses a steeper gradient and a larger slope than other electrodes. Moreover, the high‐frequency region of Nyquist diagram (insert in Figure 4k) illustrates the smallest *x* intercept for Ni‐1‐800 electrode. The Ni‐1‐800 electrode apparently shows better electrical conductivity and faster charge/ion transfer rate. Figure 4l shows the diffusion resistance (*σ*) and Na^+^ diffusion coefficient (*D*_Na_^+^) of the Ni‐1‐800, Ni‐5‐800, and Ni‐10‐800 electrodes. The Ni‐1‐800 electrode indicates the lowest σ (3.53) and the largest *D*_Na_^+^ (28.5 × 10^−10^ cm^2^ s^−1^). These results show that the Ni‐1‐800 electrode exhibits fast Na^+^ ion diffusion. In short, the Ni‐1‐800 electrode apparently possesses the highest electrical conductivity and the fastest ion diffusion rate, which is also beneficial for the ion storage and adsorption.^[^ ^62^ ^]^

### 2.3 CDI Performance

**Figure 5:**
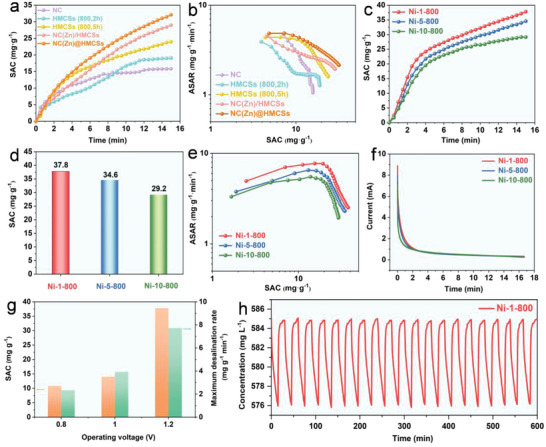
a) SAC‐time plots, and b) CDI Ragone plots of NC, HMCSs (800, 2 h), HMCSs (800, 5 h), NC(Zn)/HMCSs, and NC(Zn)@HMCSs in 10.0 × 10^−3^ m NaCl. c) SAC‐time plots, d) desalination capacities, e) CDI Ragone plots, and f) the corresponding current variation of Ni‐1‐800, Ni‐5‐800, and Ni‐10‐800 electrodes in 10.0 × 10^−3^ m NaCl. g) SAC values and maximum desalination rate of Ni‐1‐800 at different operating voltages. h) Cycle stability of Ni‐1‐800 electrode in 10.0 × 10^−3^ m NaCl solution.

The salt adsorption performances of NC, HMCSs, NC(Zn)/HMCSs, NC(Zn)@HMCSs electrodes (**Figure** **5**a) were explored in 10.0 × 10^−3^ m NaCl at 1.2 V. It can be clearly observed from SAC‐time plots that the upward trend of NC(Zn)@HMCSs is faster than that of NC(Zn)/HMCSs, HMCSs, and NC within 15 min. The corresponding SAC values (mg g^−1^) are as follows: NC(Zn)@HMCSs (32.1) > NC(Zn)/HMCSs (28.9) > HMCSs (800, 5 h) (23.9) > HMCSs (800, 2 h) (19.1) > NC (15.8), which indicates that the combination of NC(Zn) and HMCSs is conducive to the improvement of CDI characteristics. The corresponding CDI Ragone plots of these five electrodes are shown in Figure 5b. It is well known that the trend of moving up and right in these plots implies high electro‐sorption performance and rate.^[^ ^63^ ^]^ Apparently, NC(Zn)@HMCSs electrode displays the most effective adsorption efficiency during CDI process. The corresponding maximum salt adsorption rates (mg g^−1^ min^−1^) are as follows: NC(Zn)@HMCSs (4.87) > NC(Zn)/HMCSs (4.61) > HMCSs (800, 5 h) (4.37) > HMCSs (800, 2 h) (3.91). In order to better explore the effect of nickel ion doping on CDI performance, we further tested NC(Zn, Ni)@HMCSs electrodes with different nickel contents. Figure S29 (Supporting Information) shows that the concentration of NaCl solution initially sharply decreases and then tended to flatline. Correspondingly, the SAC‐time diagrams (Figure 5c) of the Ni‐1‐800, Ni‐5‐800, and Ni‐10‐800 electrodes show the fastest upward trend of Ni‐1‐800. The desalination performance of Ni‐1‐800 (37.8 mg g^−1^) can be calculated (Figure 5d), which is higher than that of Ni‐5‐800 (34.6 mg g^−1^), and Ni‐10‐800 (29.2 mg g^−1^). Therefore, the desalination performance of Ni‐1‐800 and Ni‐5‐800 is significantly better than that of NC(Zn)@HMCSs, indicating that Ni doping is beneficial for CDI performance. However, the desalination performance of Ni‐10‐800 is worse than that of NC(Zn)@HMCSs, which also confirms that excessive Ni ion doping is not conducive to the improvement of CDI performance. Compared with the CDI performance of other similar hollow carbon‐based materials (Table S4, Supporting Information), the Ni‐1‐800 electrode also performs well. In addition, it can be seen from the CDI Ragone diagram (Figure 5e) that the Ni‐1‐800 electrode shows the highest desalination capacity and rate. The maximum salt adsorption rate of Ni‐1‐800 (7.73 mg g^−1^ min^−1^) is higher than that of Ni‐5‐800 (6.51 mg g^−1^ min^−1^), and Ni‐10‐800 (5.51 mg g^−1^ min^−1^). While the desalination performance is evaluated, the corresponding current is also monitored (Figure 5f). The charge efficiency (*Λ*) of each electrode during CDI process is calculated according to the current change diagram. As shown in Figure S30 (Supporting Information), the *Λ* value of the Ni‐1‐800 electrode is higher than that of Ni‐5‐800 and Ni‐10‐800, which also indicates that its energy consumption is lower. In addition, the CDI performance of the Ni‐1‐800 electrode at different operating voltages (0.8–1.2 V) was also evaluated. It is evident that as the voltage increases, the SAC value and maximum desalination rate are both increasing (Figure 5g). Finally, the cycle stability for the desalination/regeneration property of Ni‐1‐800 electrode was also investigated. Figure 5h illustrates that the Ni‐1‐800 electrode shows no significant degradation after 20 salt adsorption‐desorption cycles, indicating its good cycling performance. After the desalination cycles, the TEM image and XRD pattern (Figures S31 and S32, Supporting Information) of Ni‐1‐800 sample are unchanged significantly, indicating its structural integrity, which further proves that Ni‐1‐800 electrode possesses good cyclic stability.

## 3 Conclusion

In summary, two different types of ZIF(M)‐derived nitrogen‐doped carbon and HMCSs nanocomposites (NC(M)/HMCSs, NC(M)@HMCSs) were synthesized through interface‐coating and space‐encapsulating design, respectively. The as‐prepared NC(M)/HMCSs and NC(M)@HMCSs with hierarchical hollow structure show unique features, such as abundant nitrogen doping, high specific surface area, and abundant meso‐/microporous pores. NC(M)@HMCSs exhibit higher electrochemical performance than NC(M)/HMCSs due to their better utilization of the internal space of hollow carbon, forming a better interconnected 3D framework. Meanwhile, the CDI performance for NC(Zn)@HMCSs (SAC: 32.1 mg g^−1^) is also significantly better than that of NC(Zn)/HMCSs, HMCSs, and NC. In addition, Ni ions doping has a certain improvement effect on the CDI performance for NC(M)@HMCSs, due to its better synergistic effect of NC(Zn, Ni) and N‐doped carbon shell. The results indicate that the SAC value of Ni‐1‐800 (37.8 mg g^−1^) with trace doping of Ni ions is higher than that of Ni‐5‐800 (34.6 mg g^−1^) and Ni‐10‐800 (29.2 mg g^−1^) with high nickel content. This work not only offers a potential CDI electrode material, but also provides a new direction for MOF nanoparticle‐driven assembly in the design of HMCSs‐based 3D framework nanostructure.

## Conflict of Interest

The authors declare no conflict of interest.

## Supporting information

Supporting Information

## Data Availability

The data that support the findings of this study are available in the Supporting Information of this article.
