# Supplementary material for: Enhanced Capacitive Deionization of Hollow Mesoporous Carbon Spheres/MOFs Derived Nanocomposites by Interface‐Coating and Space‐Encapsulating Design
Source: Adv Sci (Weinh). 2024 Aug 14;11(39):2403802. doi: 10.1002/advs.202403802 (PMC11497006; doi:10.1002/advs.202403802)
Supplement: Supplementary file 1 — Supporting Information [file ADVS-11-2403802-s001.docx]

Supporting Information

**Enhanced Capacitive Deionization of Hollow Mesoporous Carbon Spheres/MOFs Derived Nanocomposites by** **Interface-Coating and Space-Encapsulating Design**

*Yijian Tang, Yuxin Shi, Yichun Su, Shuai Cao, Jinliang Hu, Huijie Zhou, Yangyang Sun, Zheng Liu, Songtao Zhang, Huaiguo Xue, Huan Pang**

Y. Tang, Y. Shi, Y. Su, S. Cao, H. Zhou, Y. Sun, Z. Liu, S. Zhang, Prof. H. Xue, Prof. H. Pang

School of Chemistry and Chemical Engineering, Yangzhou University, Yangzhou,

Jiangsu, 225009, P. R. China

E-mail: huanpangchem@hotmail.com; panghuan@yzu.edu.cn

J. Hu

Jiangsu Yangnong Chemical Group Co. Ltd., Yangzhou 225009, P. R. China

**Experimental Section**

***Materials***

All reagents were of analytical grade and could be used without further purification. Tetraethyl orthosilicate was purchased from Shanghai Macklin Biochemical Co., Ltd. Dopamine hydrochloride (99%) was purchased from Saan Chemical Technology (Shanghai) Co., Ltd. 2-Methylimidazole (2-MeIm), sodium chloride (NaCl) was purchased from Shanghai Aladdin Biochemical Technology Co., Ltd. Manganese nitrate, cobalt nitrate, nickel nitrate, copper nitrate, zinc nitrate, NH_3_·H_2_O, ethanol, methanol, NaOH, Poly(1,1-difluoroethylene) (PVDF), N-methyl 2-pyrrolidinone (NMP) were purchased from Sinopharm Chemical Reagent Co., Ltd. Vulcan XC 72 was purchased from Cabot Corporation Agar-agar. All aqueous solutions were freshly prepared with high purity water.

***Synthesis of NC(Zn)***

Briefly, Zn (NO_3_)_2_·6H_2_O (1.0 mmol) and 2-MeIM (6.0 mmol) were dissolved in methanol (20 mL and 20 mL, resp.). The 2-MeIM solution (20 mL) was then added to the Zn (NO_3_)_2_ solution to obtain a clear solution. After stirring 2 h, the product (ZIF-8) was centrifuged and washed several times by methanol and dried. ZIF-8 was converted to nitrogen-doped carbon (NC) by carbonization at 800 °C (2 °C min^-1^) for 2 h under a N_2_ atmosphere.

***Synthesis of*** ***SiO_2_@SiO_2_+PDA***

Silica-PDA composite (SiO_2_@SiO_2_+PDA) nanospheres were synthesized using a modified stöber method. In a typical procedure, 6 mL NH_3_·H_2_O was added to a mixed solution of 140 mL ethanol and 20 mL H_2_O under stirring. Then, 5.6 mL TEOS was added and reacted under magnetic stirring for 30 min. Following this, 320 mL of an aqueous solution of dopamine (2.5 mg mL^-1^) was slowly injected into the above solution. The mixed solution was further stirred for 12 h to yield a brownish-black solid. The product was centrifuged and washed several times by water and ethanol and dried.

***Synthesis of HMCSs***

SiO_2_@SiO_2_+PDA nanospheres were converted to silica-mesoporous carbon spheres (SiO_2_@SiO_2_+MCSs) by carbonization at 800 °C (2 °C min^-1^) for 2 h under a N_2_ atmosphere. Next, the SiO_2_@SiO_2_+MCSs were completely etched in 3M NaOH for 2 days to obtain hollow mesoporous carbon spheres (HMCSs (800, 2h)). Under the same conditions, changing the above calcination time from 2 hours to 5 hours yields HMCSs (HMCSs (800, 5h)).

***Synthesis of*** ***ZIF(M)/SiO_2_@SiO_2_+PDA***

Briefly, Zn (NO_3_)_2_·6H_2_O (1.0 mmol) and SiO_2_@SiO_2_+PDA (0.1 g) were dissolved in 20 mL methanol. After ultrasonic dispersion for 10 min, the mixture solution was stirred for 2 h. Then, 20 mL of 2-MeIM methanol solution (0.3 M) was added. The mixture was stirred for 2 h. The product (marked as ZIF(Zn)/SiO_2_@SiO_2_+PDA) was centrifuged and washed several times by methanol and dried. In addition, other nitrates (0.1 mmol) were also added when Zn (NO_3_)_2_·6H_2_O and SiO_2_@SiO_2_+PDA were mixed at the beginning, such as Mn (NO_3_)_2_·4H_2_O, Co (NO_3_)_2_·6H_2_O, Ni (NO_3_)_2_·6H_2_O, and Cu (NO_3_)_2_·3H_2_O. Afterwards, the products obtained through the same steps mentioned above are labeled as ZIF(Zn, Mn)/SiO_2_@SiO_2_+PDA, ZIF(Zn, Co)/SiO_2_@SiO_2_+PDA, ZIF(Zn, Ni)/SiO_2_@SiO_2_+PDA, and ZIF(Zn, Cu)/SiO_2_@SiO_2_+PDA.

***Synthesis of*** ***NC(M)/HMCSs***

The above ZIF(M)/SiO_2_@SiO_2_+PDA nanospheres were converted to SiO_2_@carbon nanospheres (NC(M)/SiO_2_@SiO_2_+MCSs) by carbonization at 800 °C (2 °C min^-1^) for 2 h under a N_2_ atmosphere. Next, the NC(M)/SiO_2_@SiO_2_+MCSs were completely etched in 3M NaOH for 2 days to obtain NC(M)/HMCSs. The corresponding products corresponding to different doped nitrates are labeled as NC(Zn)/HMCSs, NC(Zn, Mn)/HMCSs, NC(Zn, Co)/HMCSs, NC(Zn, Ni)/HMCSs, NC(Zn, Cu)/HMCSs.

***Synthesis of* *ZIF(M)@HMCSs***

Briefly, Zn (NO_3_)_2_·6H_2_O (40.0 mg) and HMCSs (800, 5h) (10.0 mg) were dissolved in 4 mL methanol. After ultrasonic dispersion for 30 min, the mixture solution was stirred for 12 h. Then, 4 mL of 2-MeIM methanol solution (13.12 mg/mL) was added. The mixture was stirred for 2 h at a 5 ^o^C bath. The product (marked as ZIF(Zn)@HMCSs) was centrifuged and washed several times by methanol and dried. In addition, other nitrates (4.0 mg) were also added when Zn (NO_3_)_2_·6H_2_O and HMCSs (800, 5h) were mixed at the beginning, such as Mn (NO_3_)_2_·4H_2_O, Co (NO_3_)_2_·6H_2_O, Ni (NO_3_)_2_·6H_2_O, and Cu (NO_3_)_2_·3H_2_O. Afterwards, the products obtained through the same steps mentioned above are labeled as ZIF(Zn, Mn)@HMCSs, ZIF(Zn, Co)@HMCSs, ZIF(Zn, Ni)@HMCSs, and ZIF(Zn, Cu)@HMCSs.

***Synthesis of* *NC(M)@HMCSs***

The above ZIF(M)@HMCSs nanospheres were converted to HMCSs/ZIFs-derived carbon materials (NC(M)@HMCSs) by carbonization at 800 °C (2 °C min^-1^) for 2 h under a N_2_ atmosphere. The corresponding products corresponding to different doped nitrates are labeled as NC(Zn)@HMCSs, NC(Zn, Mn)@HMCSs, NC(Zn, Co)@HMCSs, NC(Zn, Ni)@HMCSs, NC(Zn, Cu)@HMCSs.

***Synthesis of* *Ni-x-800***

When preparing ZIF(Zn, Ni)@HMCSs samples, changing the amount of Ni (NO_3_)_2_·6H_2_O doped (4.0 mg, 20.0 mg, 40.0 mg), the resulting products were labeled as Ni-1, Ni-5, and Ni-10. The above Ni-*x* samples were converted to NC(Zn, Ni)@HMCSs by carbonization at 800 °C (2 °C min^-1^) for 2 h under a N_2_ atmosphere. The corresponding obtained products are labeled as Ni-1-800, Ni-5-800, and Ni-10-800.

***Materials characterization***

The morphological features were characterized by field emission scanning electron microscopy (FESEM, Zeiss-Supra55), high resolution transmission electron microscopy (HRTEM, Tecnai G2 F30 S-TWIN), and energy dispersive X-ray spectrometry (EDS) mapping. X-ray diffraction (XRD) patterns were examined on a Bruker D8 Advanced X-ray Diffractometer (Cu-Kα radiation: λ = 0.15406 nm). Raman spectroscopy was obtained by using Renishaw InVia Reflex (514 nm laser). The chemical states are measured using an Axis Ultra X-ray photoelectron spectroscope (XPS, Kratos Analytical Ltd., UK) equipped with a standard monochromatic Al-Kα source (hv = 1486.6 eV). Fourier transform infrared (FT-IR) transmission spectra were obtained on a BRUKER-EQUINOX-55 IR spectrophotometer. Nitrogen sorption isotherms were carried out using a BELSORP-mini (BEL, Japan). The specific surface area (SSA) was analyzed by Multipoint Brunauer-Emmett-Teller (BET) technique.

***Electrochemical performance measurements***

The electrode ink was prepared by mixing 80 wt% active material with 10 wt% Vulcan XC 72 and 10 wt% PVDF in NMP solvent under ultrasonication for 30 min. A certain volume of the ink was dropped onto the graphite paper with a thickness of 0.5 mm (area of 1 × 1 cm^2^) and dried at 60 °C for 12 h. The potential sweep cyclic voltammetry (CV) and electrochemical impedance spectroscopy (EIS) measurements were conducted by using an electrochemical workstation (CHI-760E) with three-electrode configuration in 1.0 M NaCl electrolyte. The Ag/AgCl electrode and platinum (Pt) wire were used as reference and counter electrodes, respectively. Cyclic voltammetry (CV) and gravimetric charge-discharge (GCD) measurements were carried out in the potential range of -0.5 to 0.5 V.

The specific capacitance of electrodes was calculated from CV using Eq. (S1),

***C*_m_ = (∫*I*d*V*)/2*v* ∆*Vm*** **Eq. (S1-1)**

where *C_m_* (F g^-1^) represents for the specific capacitance of the electrode, *I* (A) for the current density, ∆*V* for the voltage change, *v* (mV s^-1^) for the scan rate and *m* (g) for the mass of the working electrode.

Specific capacitances (C, F g^-1^) were calculated from the discharge curves by using the following equation:


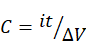
 **Eq. (S1-2)**

where
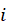
 is the discharge current density (A g^−1^), t is the discharge time (s), and ΔV is the voltage window (V).

The kinetic of capacitive behavior were also analyzed with CV measurements. Conventionally, the scan rate and the measured current obeyed the following relationship:

***i = av^b^* Eq. (S2)**

***logi = blogν + loga*** **Eq. (S3)**

where *i* presented the current; *v* denoted the scan rate; *a* and *b* expressed empirical parameters.

Furthermore, the total capacitive contribution at a given scan rate could be distinguished into two parts: the diffusion-controlled process (***k_2_v^1/2^***) and capacitive effect (***k_1_v***) at a fixed potential (V) according to the following transformed equation:

***i(V)*** ***=*** ***k_1_v +*** ***k_2_v^1/2^*** **Eq. (S4)**

There, *k_1_* and *k_2_* can be facilely achieved by linearly fitting *i(V)/v^1/2^* vs *v^1/2^*, and thus the capacitive current *ic(V) = k_1_v* could be extracted from the total one with the value of *k_2_*.

The diffusion coefficient (D_Na_^+^, cm^2^ s^-1^) was calculated by the following equation

***D_Na_^+^ = 0.5R^2^T^2^/c^2^F^4^A^2^σ^2^* Eq. (S5)**

***Z’ = σw^-1/2^* Eq. (S6)**

where *R* is the gas constant, *T* is the absolute temperature, *c* is the concentration of Na^+^ in the material, *F* is the Faraday constant, *A* is the surface area of the electrode, *σ* is the Warburg factor, and *Z’* is the real part impedance.

***CDI performance measurements***

Membrane assisted CDI (MCDI) unit-cell was constructed with two pairs of identical electrodes, ion exchange membrane for anion and cation, and spacer. Anion- and cation exchange membranes were used to alleviate the co-ion effect. Each individual CDI carbon electrode was composed of active material, Vulcan XC 72, and PVDF in the ratio of 8:1:1, and prepared on the titanium plate of 2×2 cm^2^ (thickness: 1 mm) as current collector. The mass loading of CDI carbon-based material on the titanium plate is ≈2.5 mg cm^-2^. Before assembling the MCDI unit-cell, the electrodes were immersed in 584 mg/L of saline water for 24 h to completely wet the surface. The CDI tests were conducted using a batch-mode with a continuous recycling system, which includes a CDI cell, a peristaltic pump, a power source, and a fluid reservoir. The ambient temperature and the total volume of the NaCl solution in the desalination experiment were maintained at 298 K and 50 mL, respectively. In the CDI desalination process, the saline water was desalinated through MCDI unit-cell and recycled in a closed circuit. The real-time change of the brackish water concentration was measured by a conductivity probe which was connected to the CDI system. The correlation between conductivity and concentration was achieved based on a calibration table prepared before the test (**Figure S33**). The applied voltage at both ends of the electrodes is 0.8, 1.0, 1.2 V, respectively. The salt adsorption capacity (SAC, mg g^-1^) and average salt adsorption rates (ASAR, mg g^-1^ min^-1^) at t min were calculated as follows:


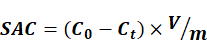
**Eq. (S7)**


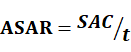
 **Eq. (S8)**

where *C*0 and *C*t are the NaCl concentrations at initial stage and *t* min, respectively (mg L^-1^), *V* is the volume of the NaCl solution (L), and *m* is the total mass of the electrode materials (g).

The charge efficiency (*Λ*) was calculated according to **Eq. (S9)**:


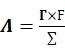
 **Eq. (S9)**

where Γ (mol g^-1^) is the desalination capacity, *F* is the Faraday constant (96485 C mol^-1^), and ∑ (charge, C g^-1^) is obtained by integrating current.


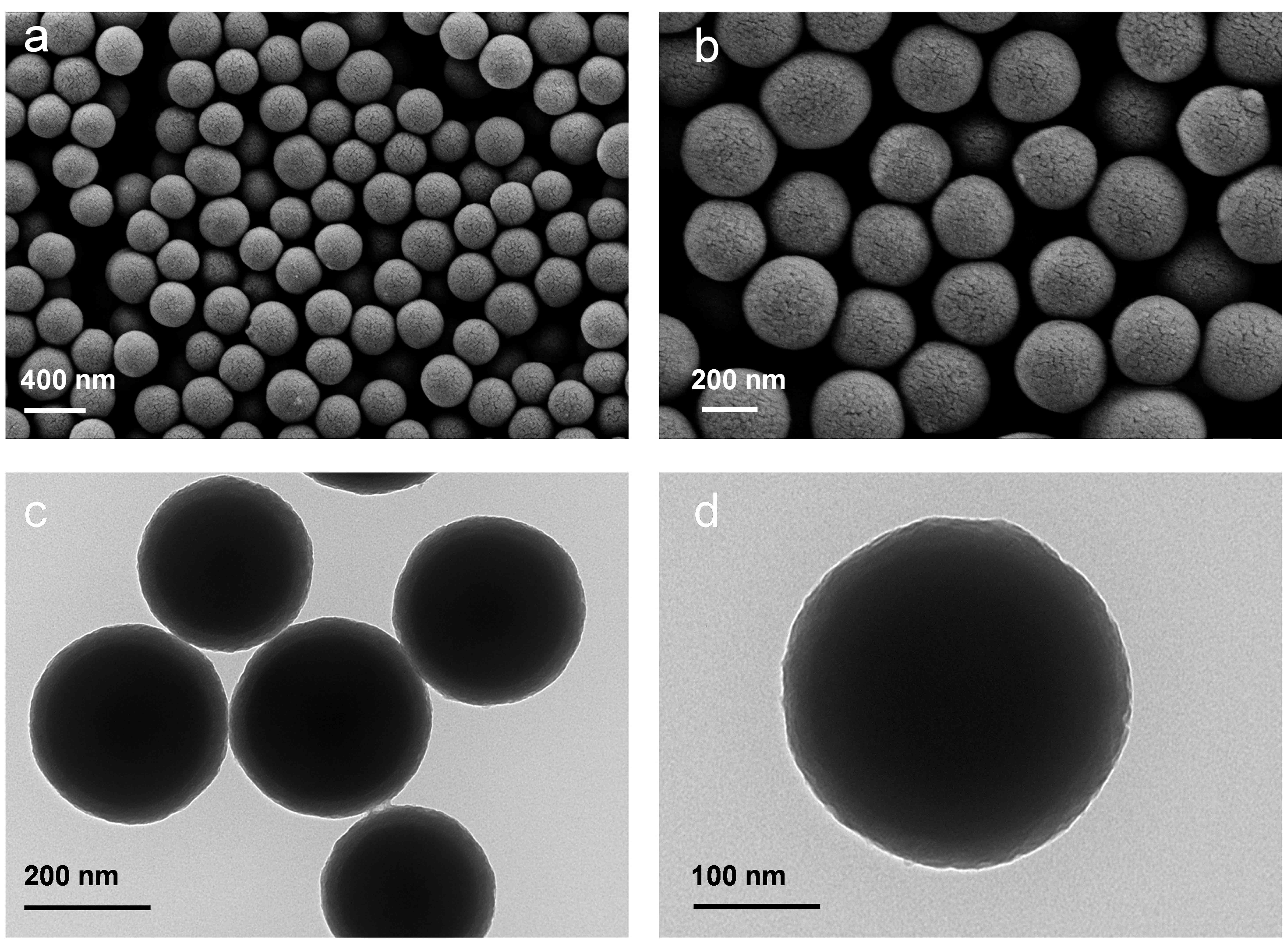


**Figure S1.** (a, b) SEM images of SiO_2_@SiO_2_+PDA. (c, d) TEM images of SiO_2_@SiO_2_+PDA.

**
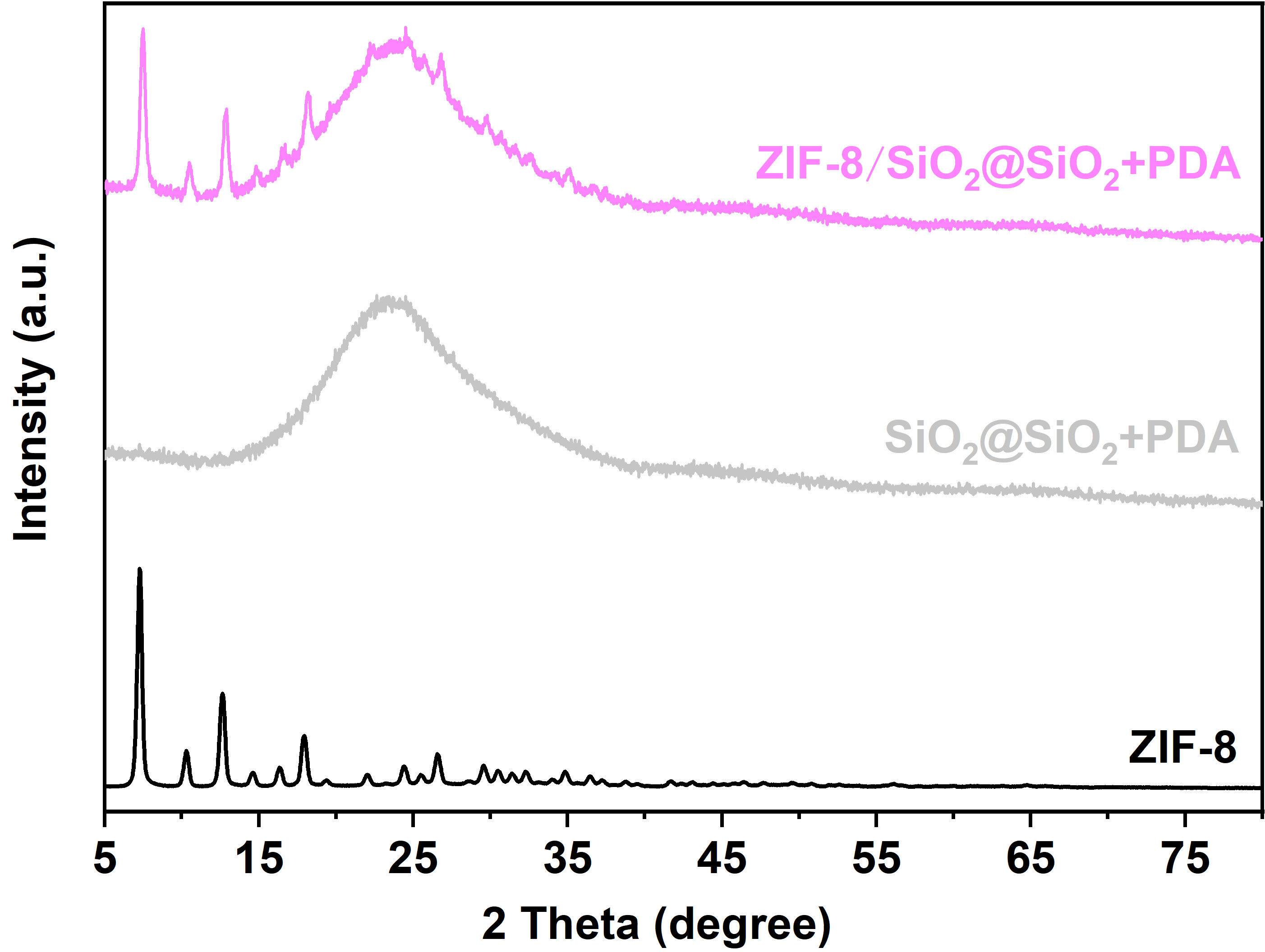
**

**Figure S2.** XRD patterns of ZIF-8, SiO_2_@SiO_2_+PDA, ZIF-8/ SiO_2_@SiO_2_+PDA.


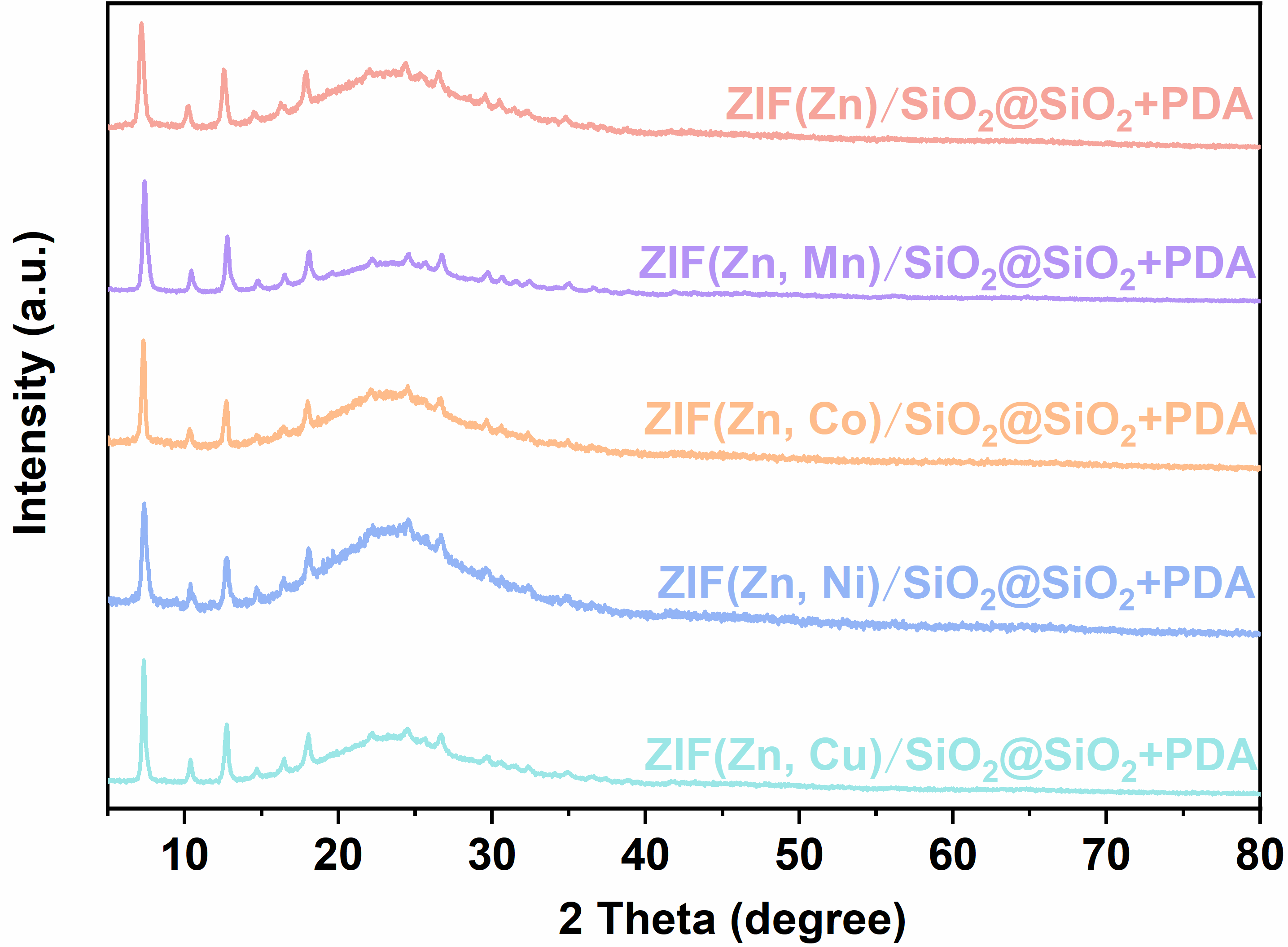


**Figure S3.** XRD patterns of ZIF(Zn)/SiO_2_@SiO_2_+PDA, ZIF(Zn, Mn)/SiO_2_@SiO_2_+PDA, ZIF(Zn, Co)/SiO_2_@SiO_2_+PDA, ZIF(Zn, Ni)/SiO_2_@SiO_2_+PDA, ZIF(Zn, Cu)/SiO_2_@SiO_2_+PDA.


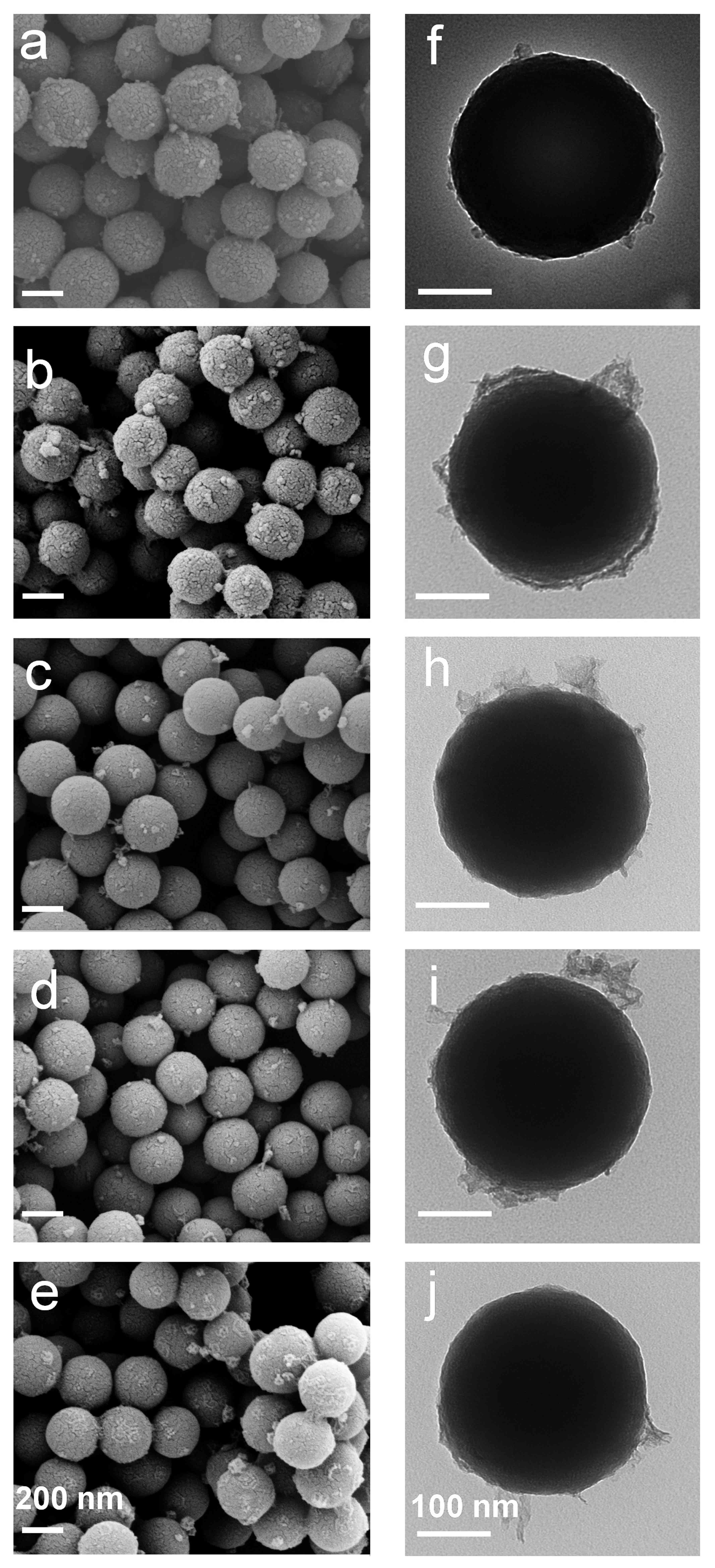


**Figure S4.** SEM images of (a) NC(Zn)/SiO_2_@SiO_2_+MCSs, (b) NC(Zn, Mn)/SiO_2_@SiO_2_+MCSs, (c) NC(Zn, Co)/SiO_2_@SiO_2_+MCSs, (d) NC(Zn, Ni)/SiO_2_@SiO_2_+MCSs, (e) NC(Zn, Cu)/SiO_2_@SiO_2_+MCSs. TEM images of (f) NC(Zn)/SiO_2_@SiO_2_+MCSs, (g) NC(Zn, Mn)/SiO_2_@SiO_2_+MCSs, (h) NC(Zn, Co)/SiO_2_@SiO_2_+MCSs, (i) NC(Zn, Ni)/SiO_2_@SiO_2_+MCSs, (j) NC(Zn, Cu)/SiO_2_@SiO_2_+MCSs.


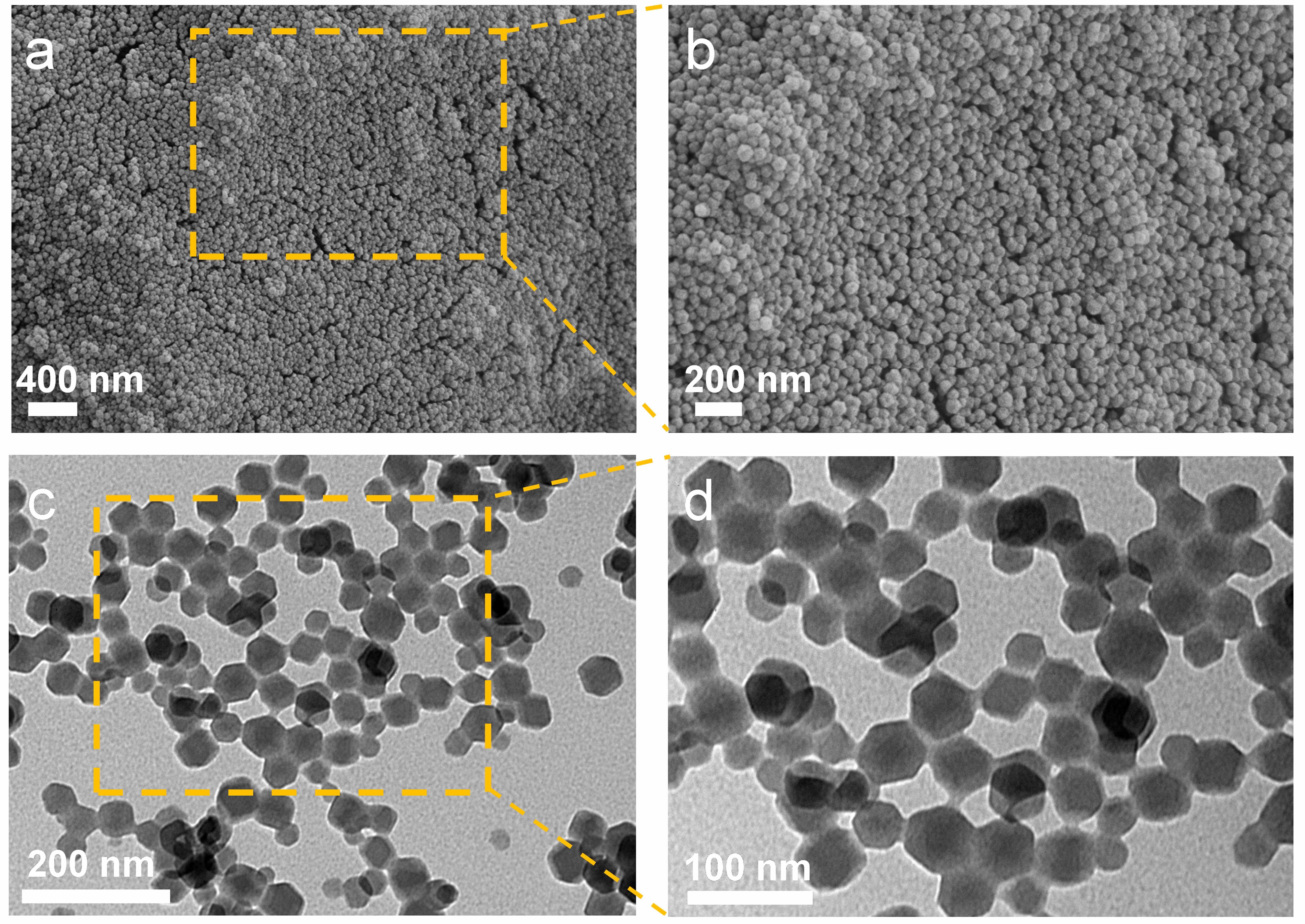


**Figure S5.** (a, b) SEM images of ZIF-8 (ZIF(Zn)). (c, d) TEM images of ZIF-8 (ZIF(Zn)).


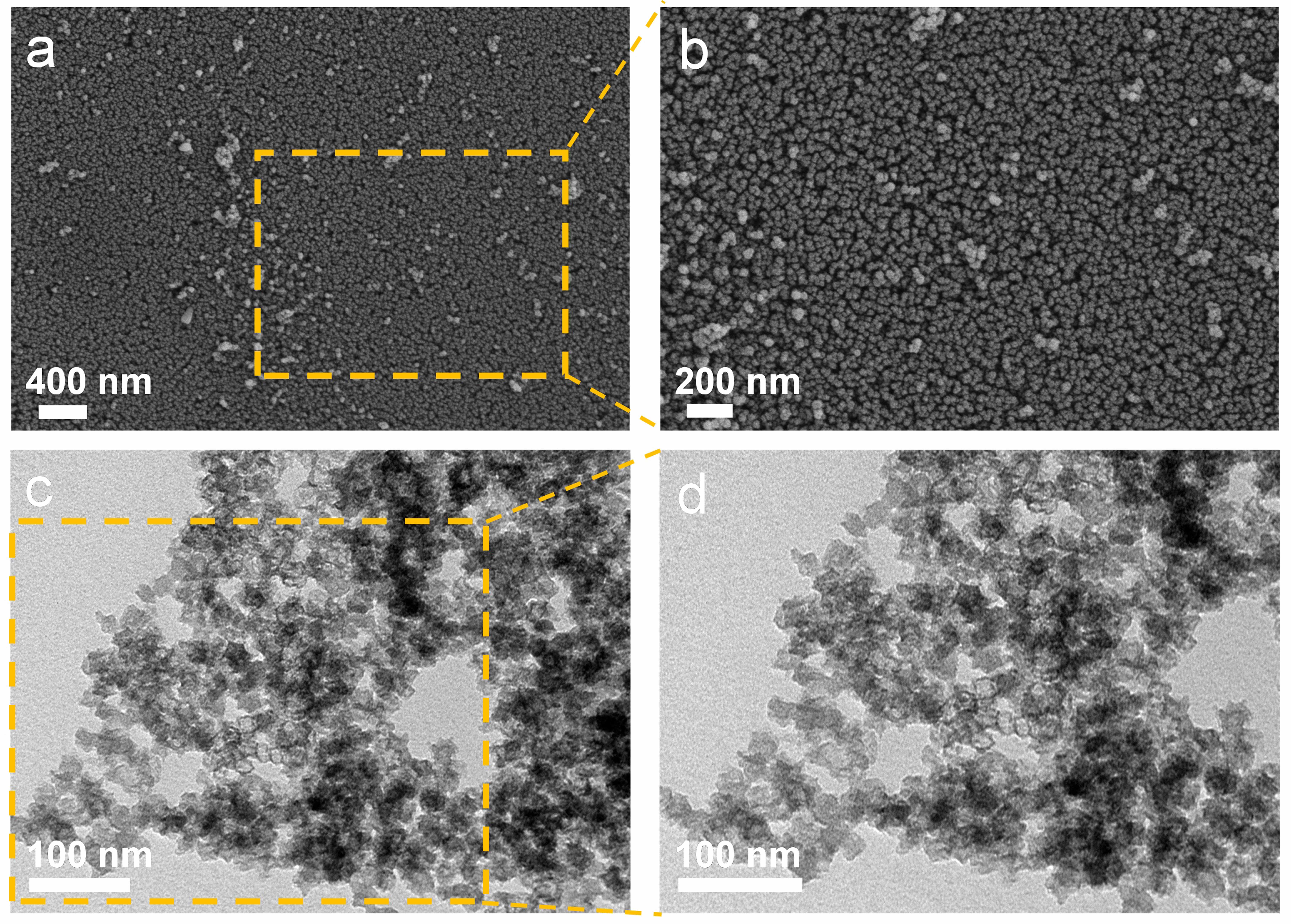


**Figure S6.** (a, b) SEM images of NC(Zn). (c, d) TEM images of NC(Zn).


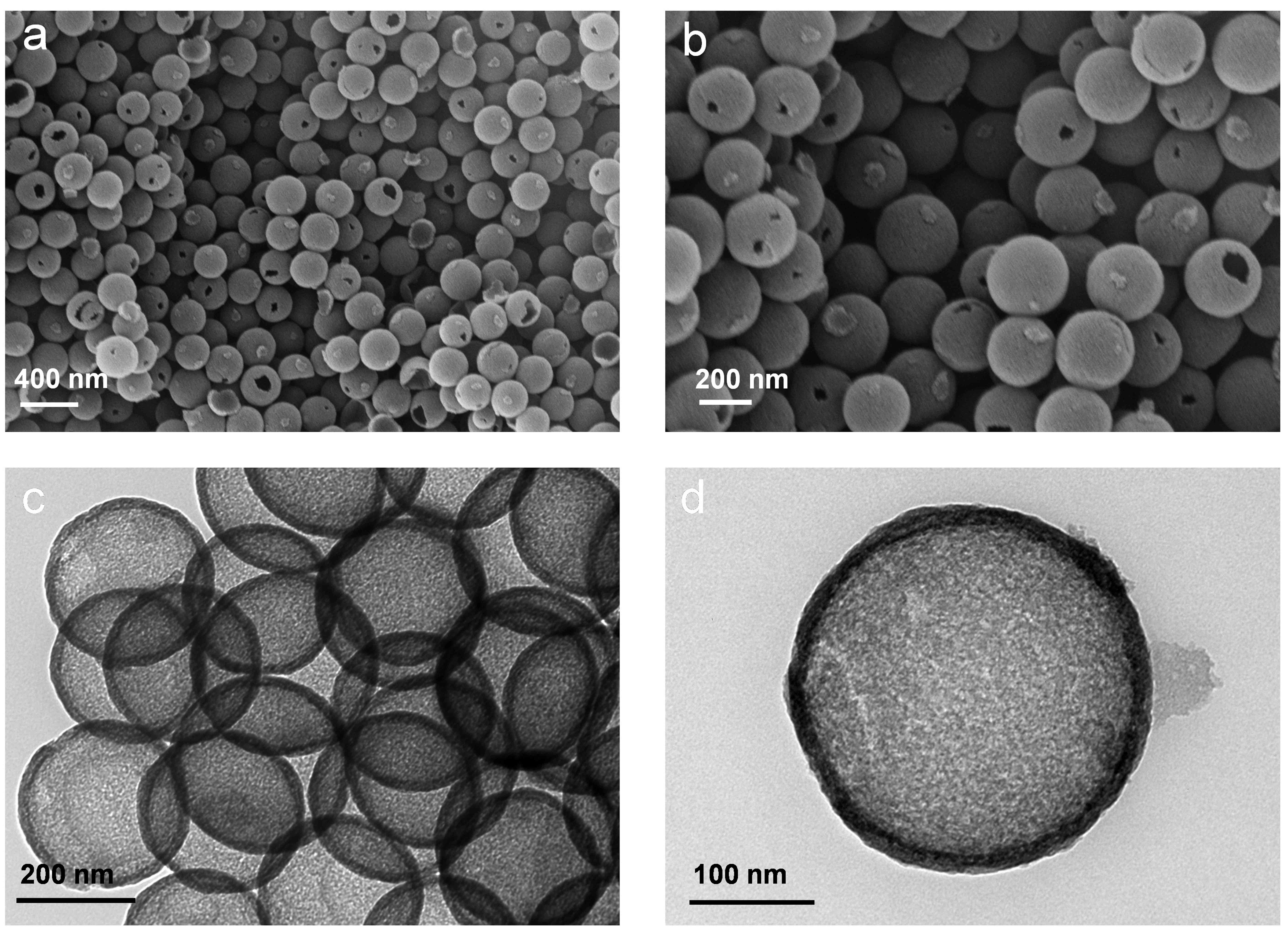


**Figure S7.** (a, b) SEM images of HMCSs (800, 5h). (c, d) TEM images of HMCSs (800, 5h).


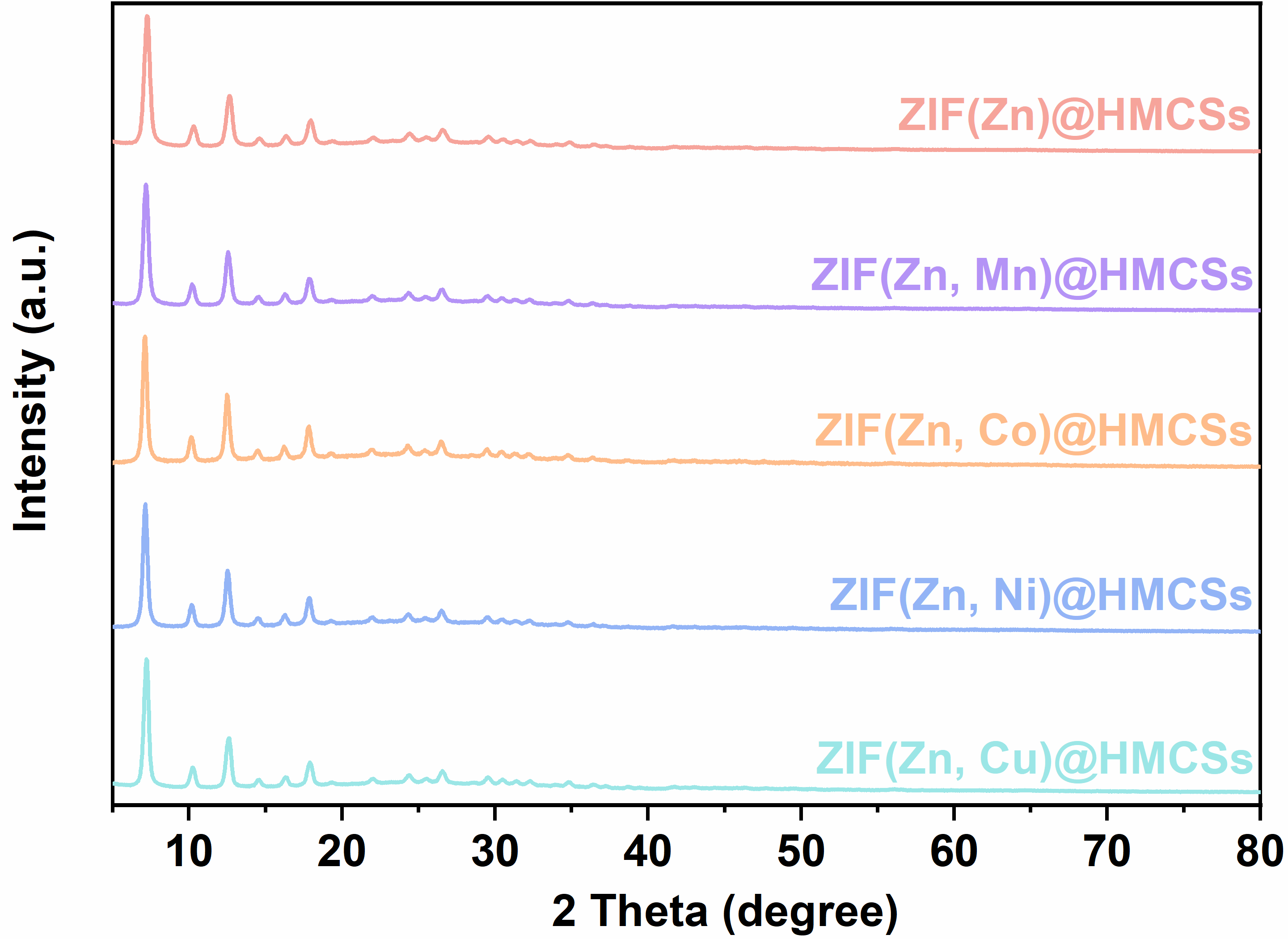


**Figure S8.** XRD patterns of ZIF(Zn)@HMCSs, ZIF(Zn, Mn)@HMCSs, ZIF(Zn, Co)@HMCSs, ZIF(Zn, Ni)@HMCSs, ZIF(Zn, Cu)@HMCSs.


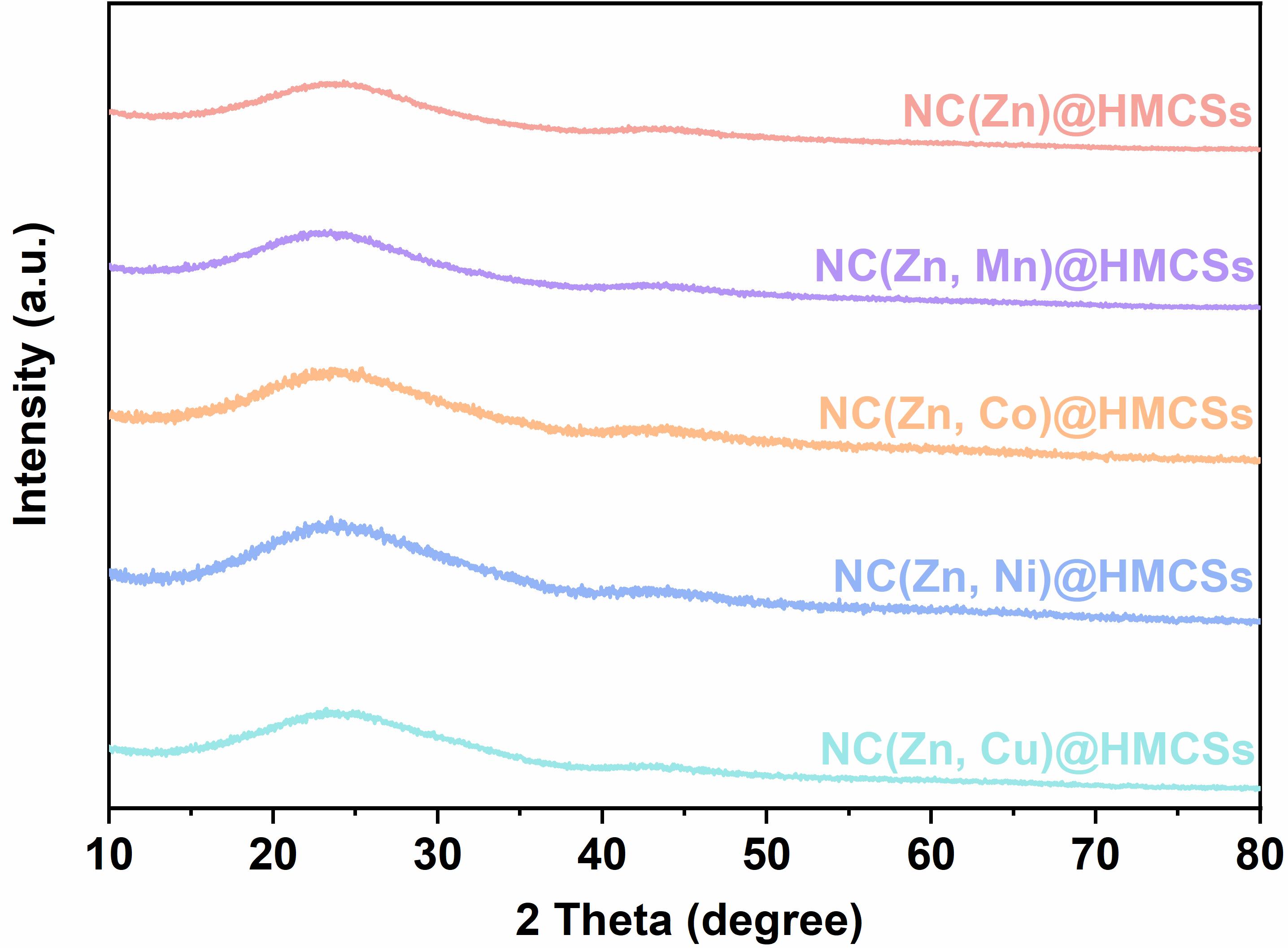


**Figure S9.** XRD patterns of NC (Zn)@HMCSs, NC (Zn, Mn)@HMCSs, NC (Zn, Co)@HMCSs, NC (Zn, Ni)@HMCSs, and NC (Zn, Cu)@HMCSs.


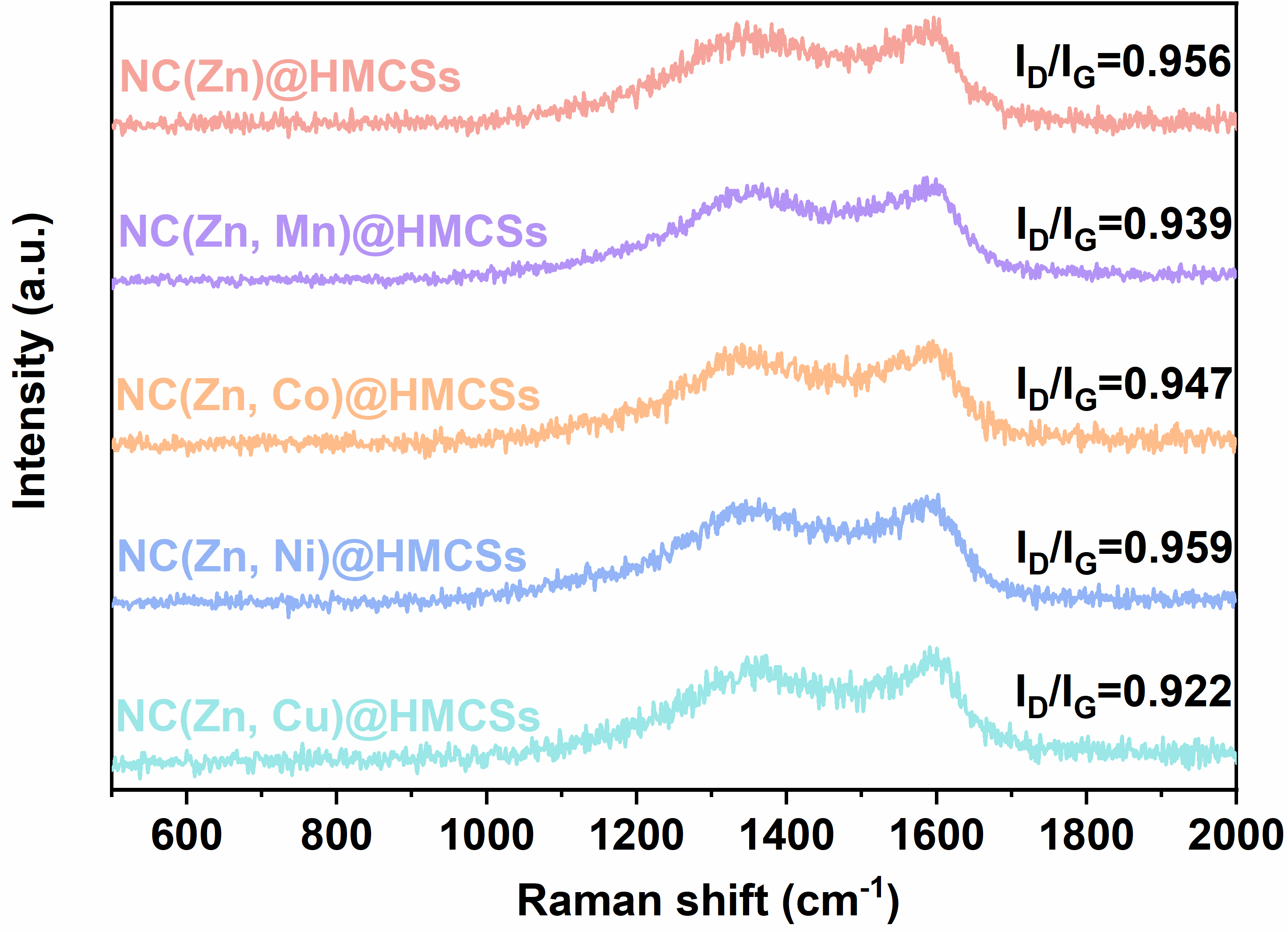


**Figure S10.** Raman spectrums of NC(Zn)@HMCSs, NC (Zn, Mn)@HMCSs, NC (Zn, Co)@HMCSs, NC (Zn, Ni)@HMCSs, and NC (Zn, Cu)@HMCSs.


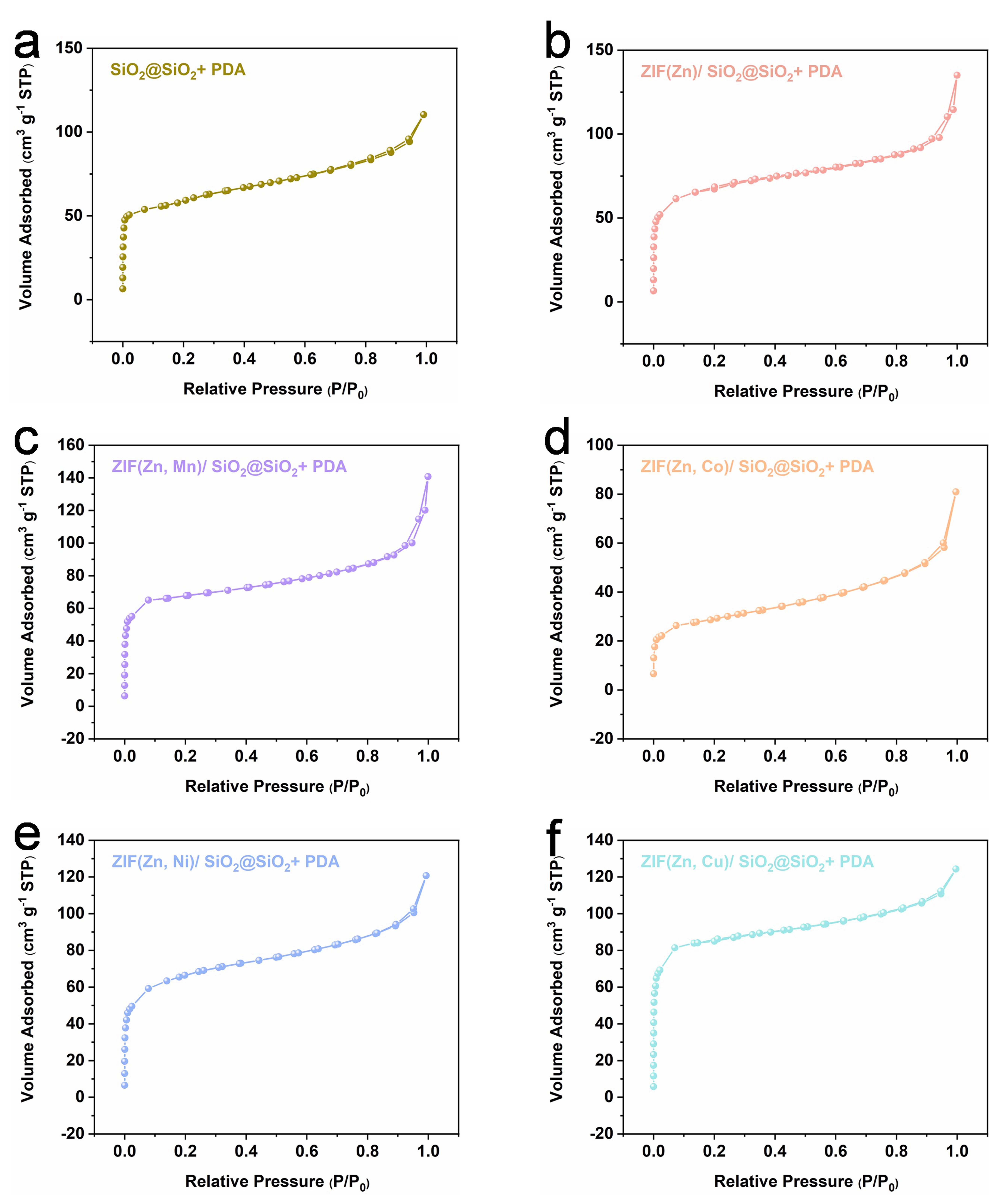


**Figure S11.** N_2_ adsorption-desorption isotherms of (a) SiO_2_@SiO_2_+PDA, (b) ZIF(Zn)/SiO_2_@SiO_2_+PDA, (c) ZIF(Zn, Mn)/SiO_2_@SiO_2_+PDA, (d) ZIF(Zn, Co)/SiO_2_@SiO_2_+PDA, (e) ZIF(Zn, Ni)/SiO_2_@SiO_2_+PDA, and (f) ZIF(Zn, Cu)/SiO_2_@SiO_2_+PDA.


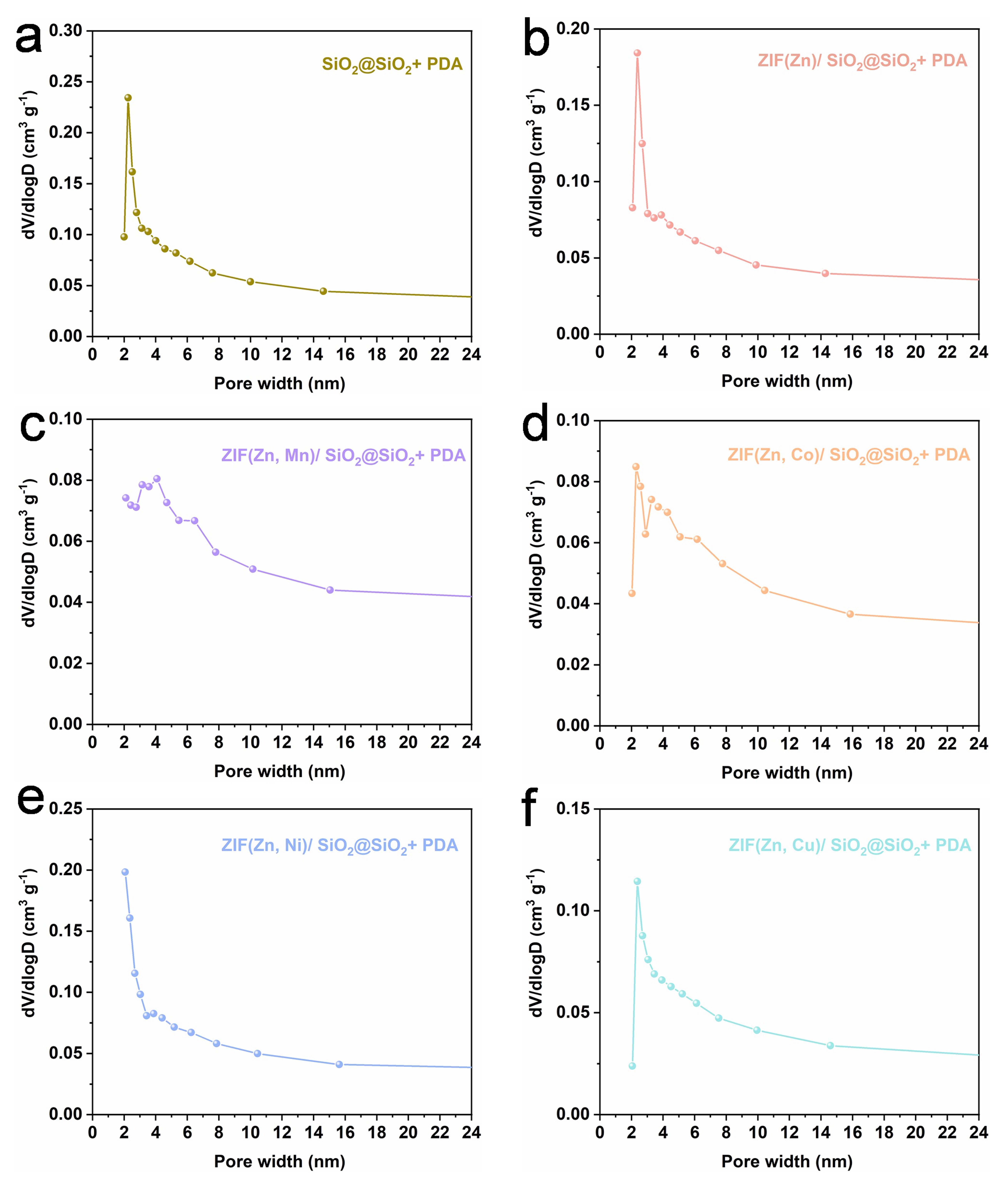


**Figure S12.** BJH pore size distribution of (a) SiO_2_@SiO_2_+PDA, (b) ZIF(Zn)/SiO_2_@SiO_2_+PDA, (c) ZIF(Zn, Mn)/SiO_2_@SiO_2_+PDA, (d) ZIF(Zn, Co)/SiO_2_@SiO_2_+PDA, (e) ZIF(Zn, Ni)/SiO_2_@SiO_2_+PDA, and (f) ZIF(Zn, Cu)/SiO_2_@SiO_2_+PDA.


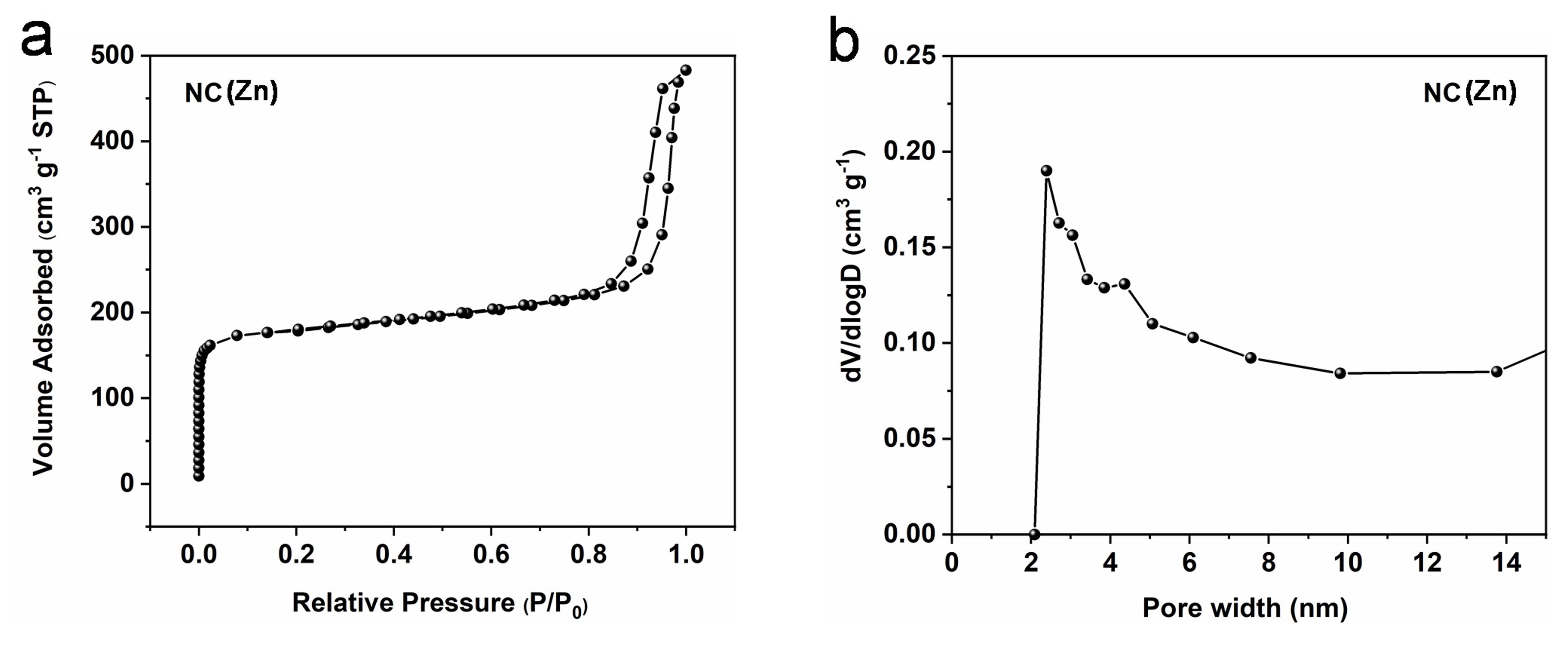


**Figure S13.** (a) N_2_ adsorption-desorption isotherms, (b) BJH pore size distribution of NC(Zn).


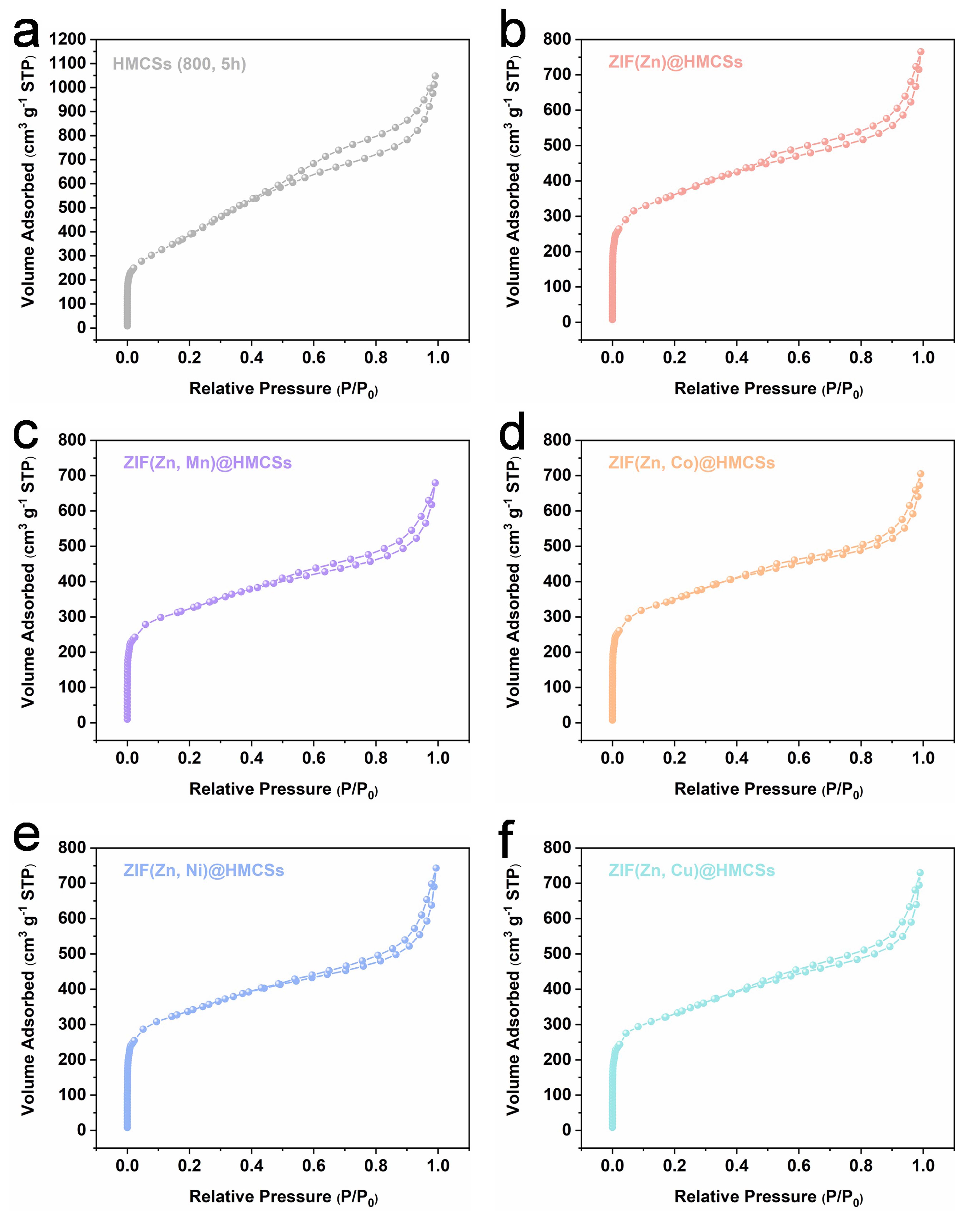


**Figure S14.** N_2_ adsorption-desorption isotherms of (a) HMCSs (800, 5h), (b) ZIF(Zn)@HMCSs, (c) ZIF(Zn, Mn)@HMCSs, (d) ZIF(Zn, Co)@HMCSs, (e) ZIF(Zn, Ni)@HMCSs, and (f) ZIF(Zn, Cu)@HMCSs.


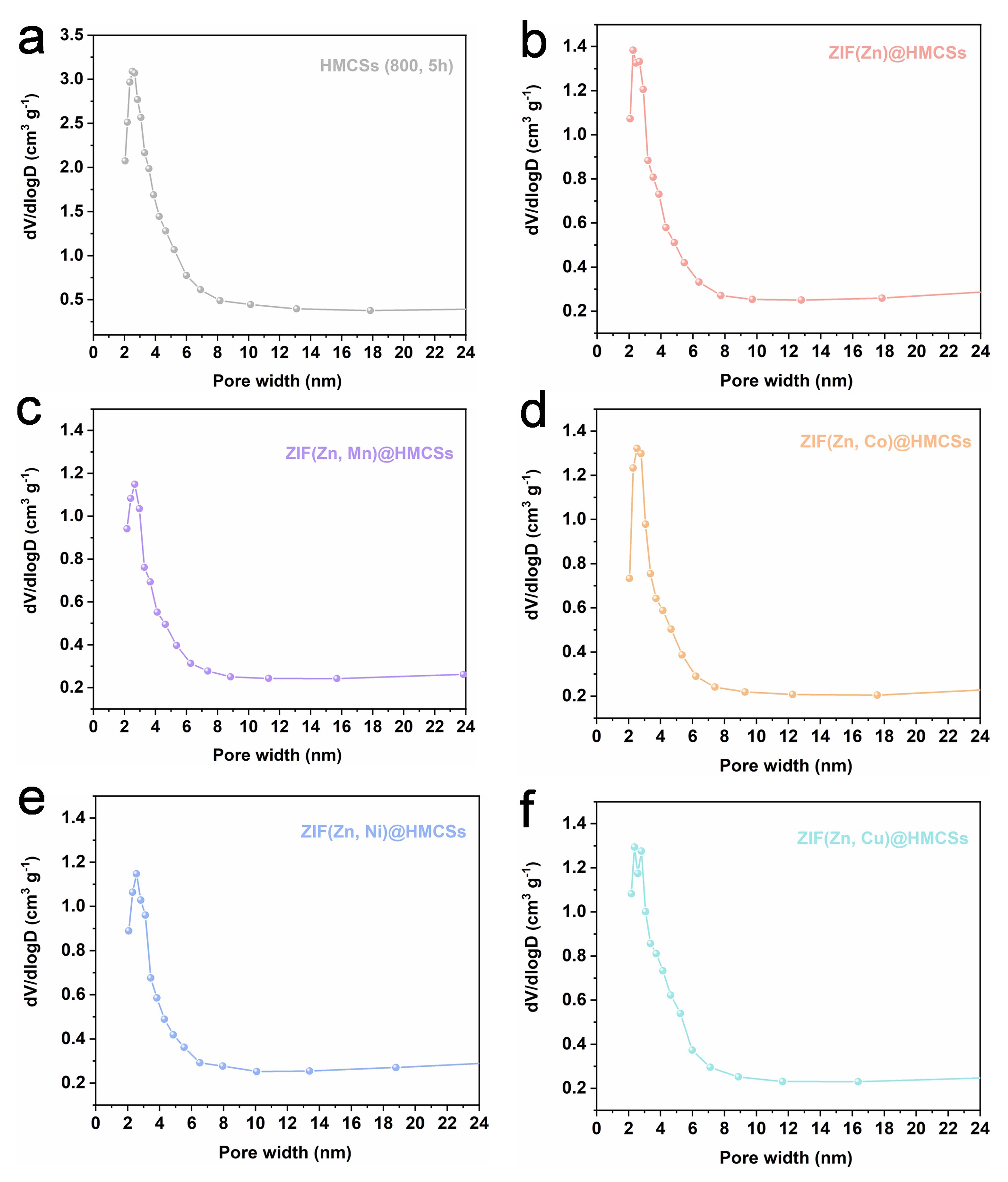


**Figure S15.** BJH pore size distribution of (a) HMCSs (800, 5h), (b) ZIF(Zn)@HMCSs, (c) ZIF(Zn, Mn)@HMCSs, (d) ZIF(Zn, Co)@HMCSs, (e) ZIF(Zn, Ni)@HMCSs, and (f) ZIF(Zn, Cu)@HMCSs.


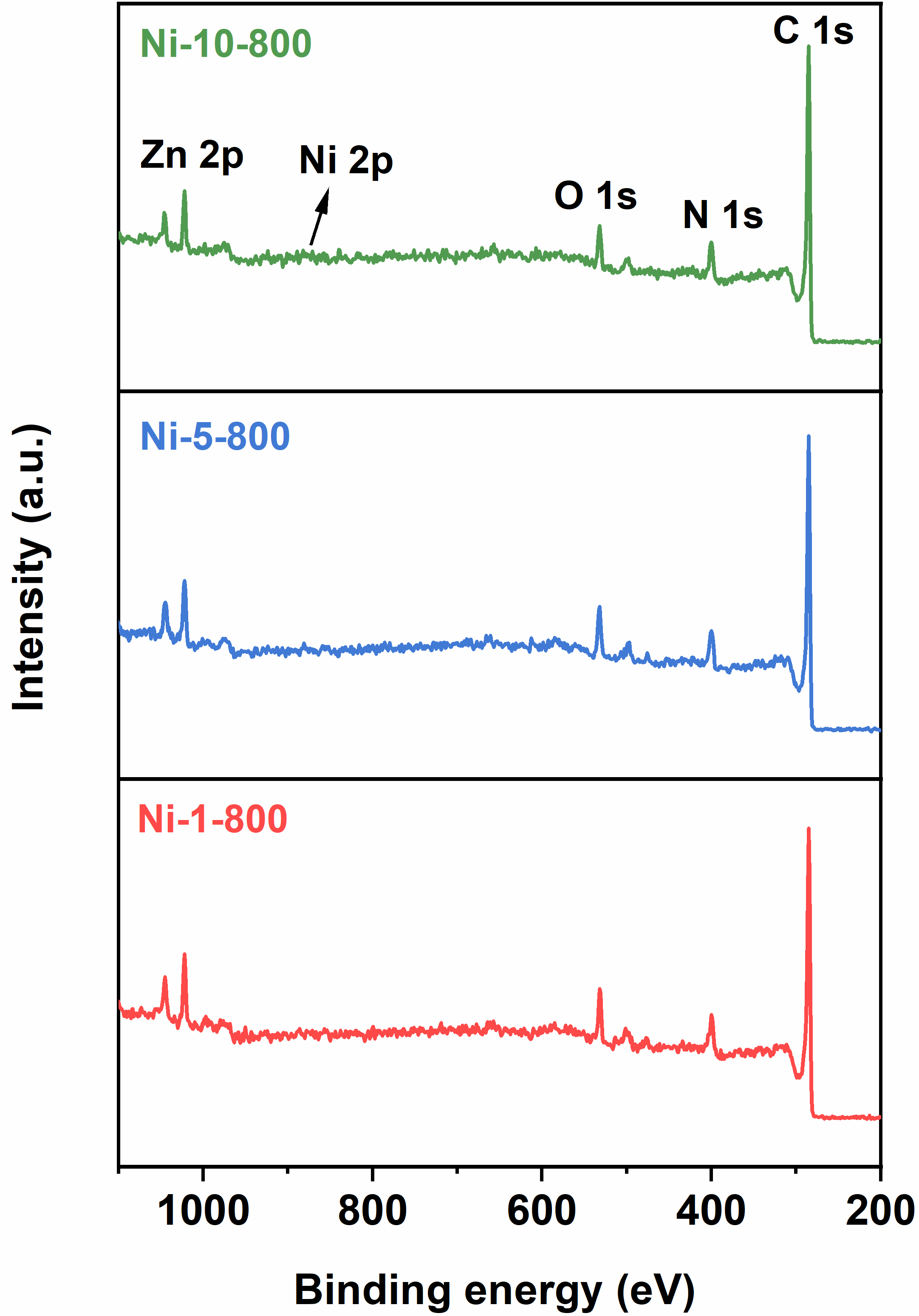


**Figure S16.** XPS survey spectra of the as-prepared Ni-1-800, Ni-5-800, Ni-10-800 samples.


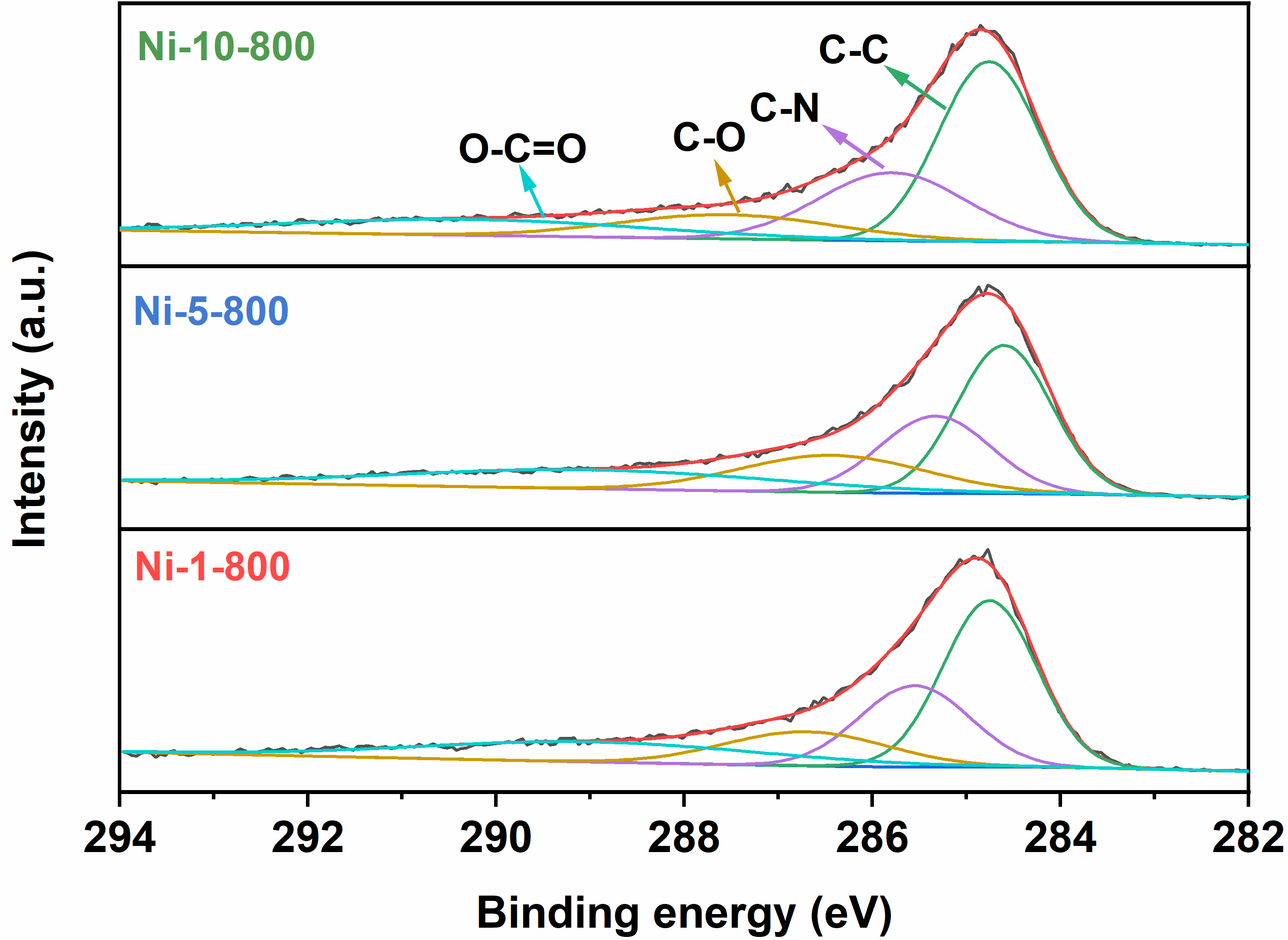


**Figure S17.** High-resolution XPS of C 1s spectra of Ni-1-800, Ni-5-800, Ni-10-800 samples.


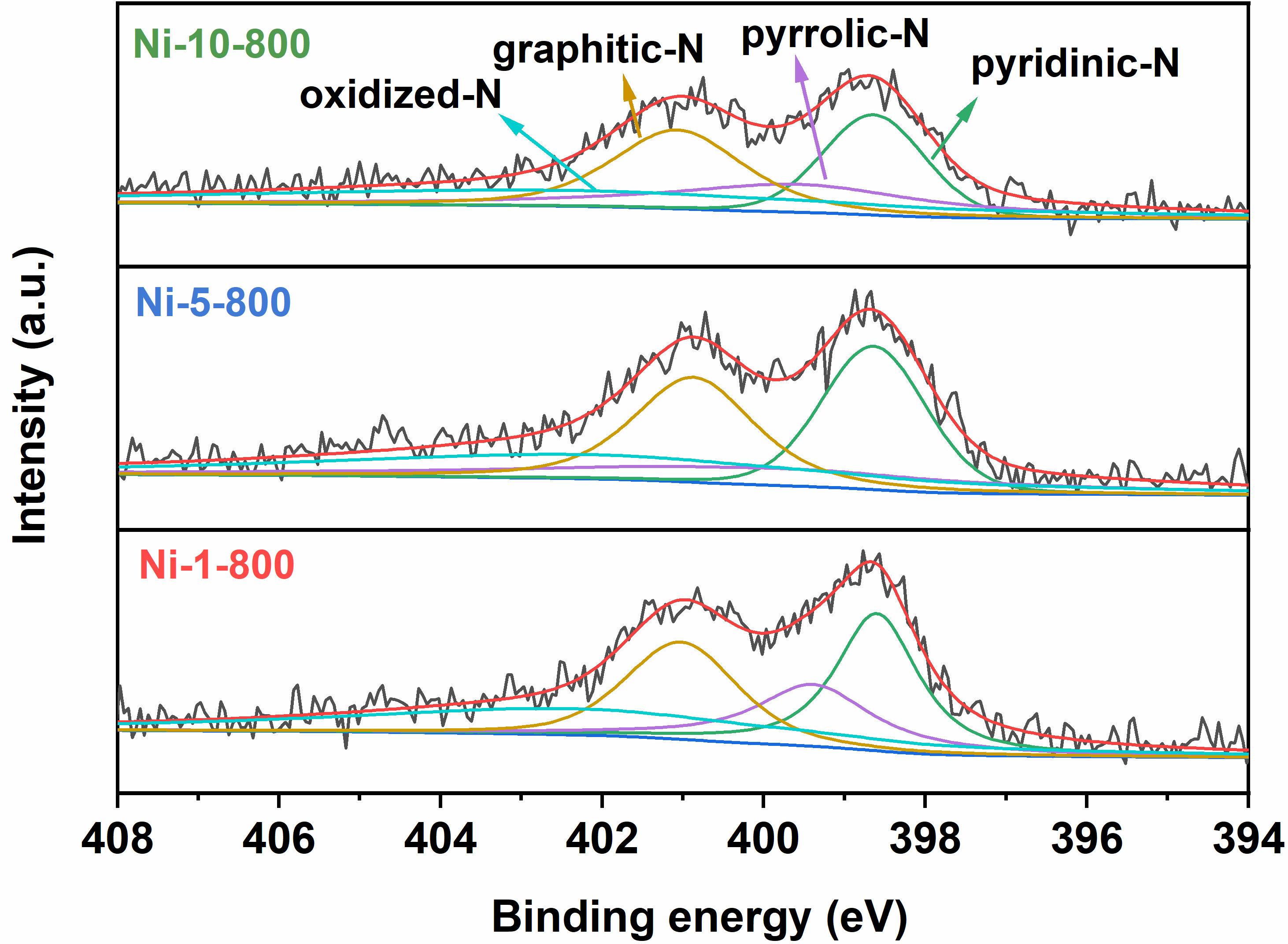


**Figure S18.** High-resolution XPS of N 1s spectra of Ni-1-800, Ni-5-800, Ni-10-800 samples.


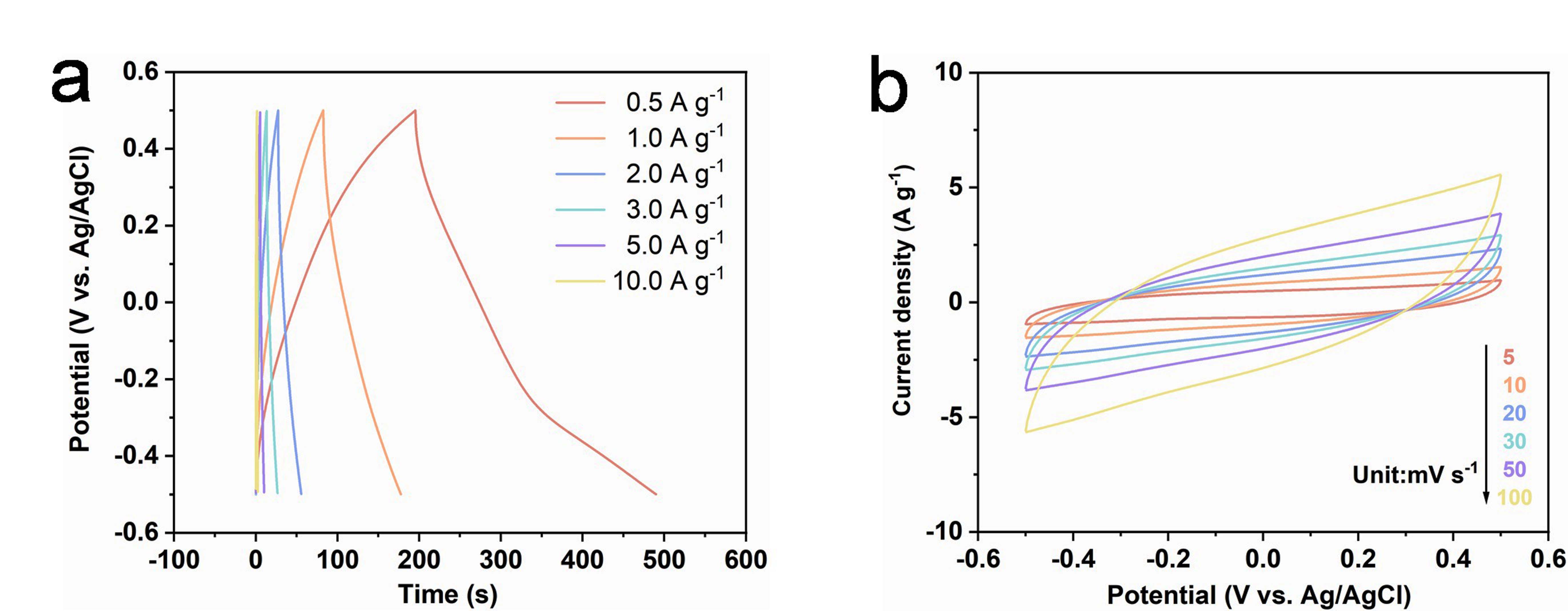


**Figure S19.** (a) GCD curves of NC(Zn) at different current densities, (b) CV curves of NC(Zn) at different scan rates.


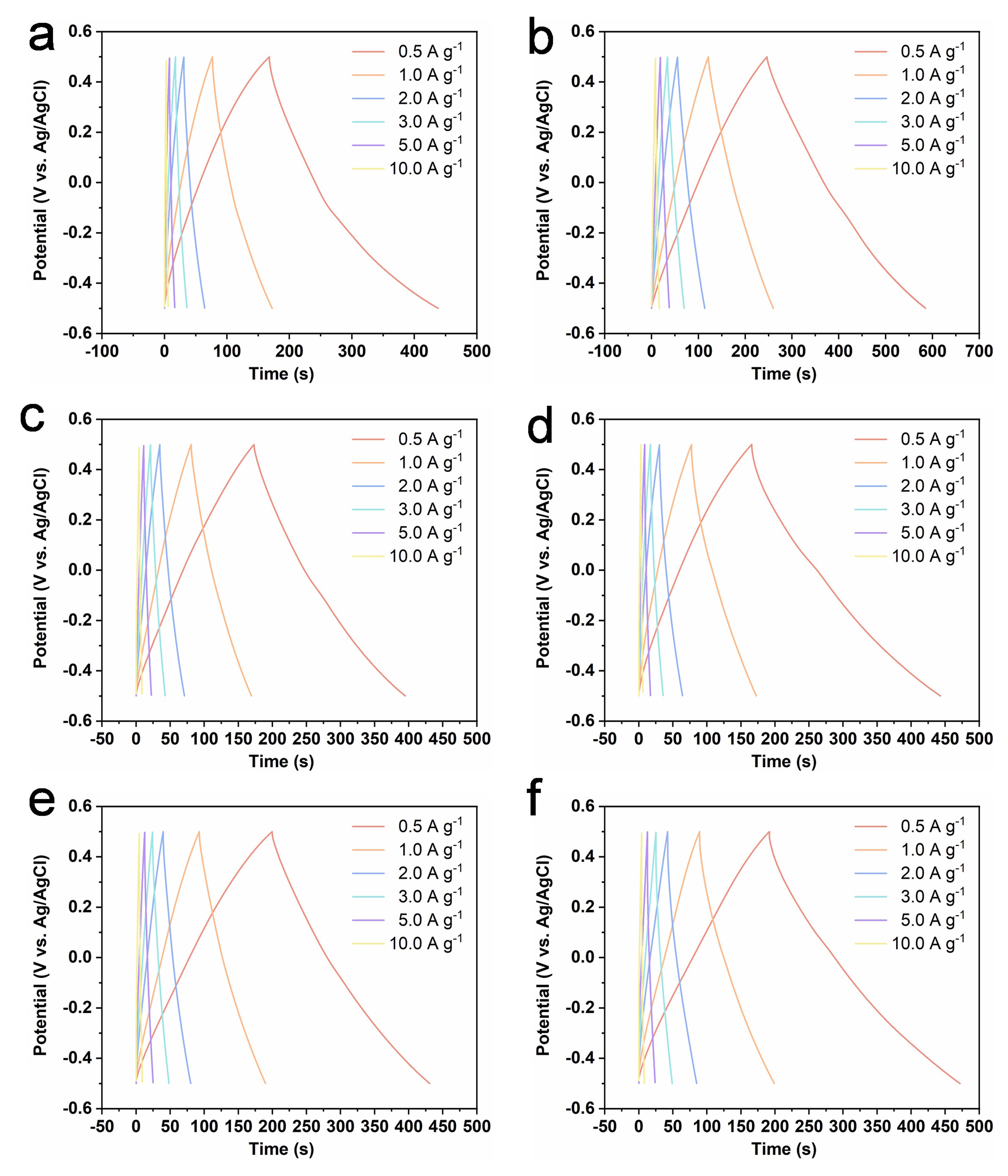


**Figure S20.** GCD curves of (a) HMCSs (800, 2h), (b) NC(Zn)/HMCSs, (c) NC(Zn, Mn)/HMCSs, (d) NC(Zn, Co)/HMCSs, (e) NC(Zn, Ni)/HMCSs, (f) NC(Zn, Cu)/HMCSs at different current densities.


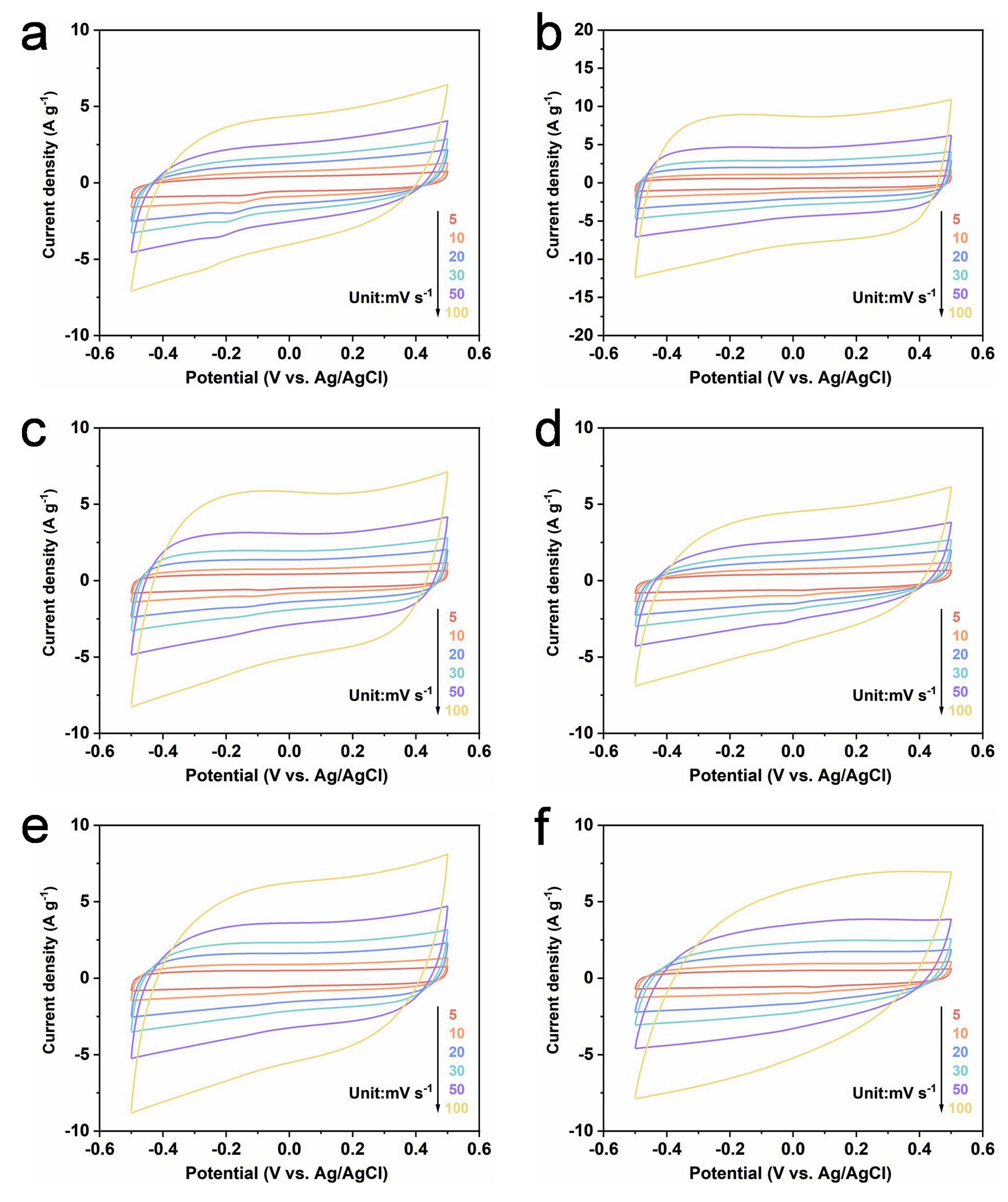


**Figure S21.** CV curves of (a) HMCSs (800, 2h), (b) NC(Zn)/HMCSs, (c) NC(Zn, Mn)/HMCSs, (d) NC(Zn, Co)/HMCSs, (e) NC(Zn, Ni)/HMCSs, (f) NC(Zn, Cu)/HMCSs at different scan rates.


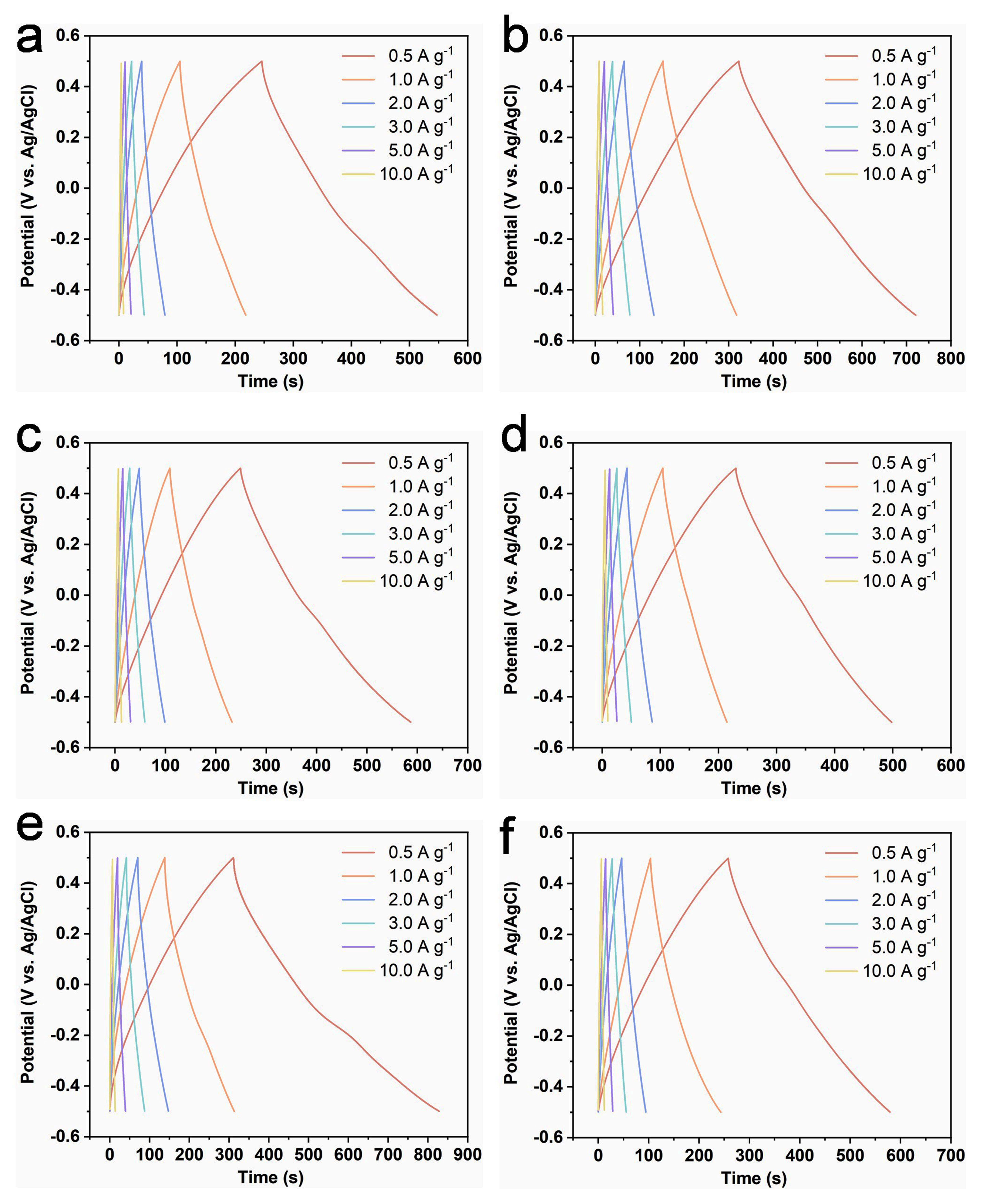


**Figure S22.** GCD curves of (a) HMCSs (800, 5h), (b) NC(Zn)@HMCSs, (c) NC(Zn, Mn)@HMCSs, (d) NC(Zn, Co)@HMCSs, (e) NC(Zn, Ni)@HMCSs, (f) NC(Zn, Cu)@HMCSs at different current densities.


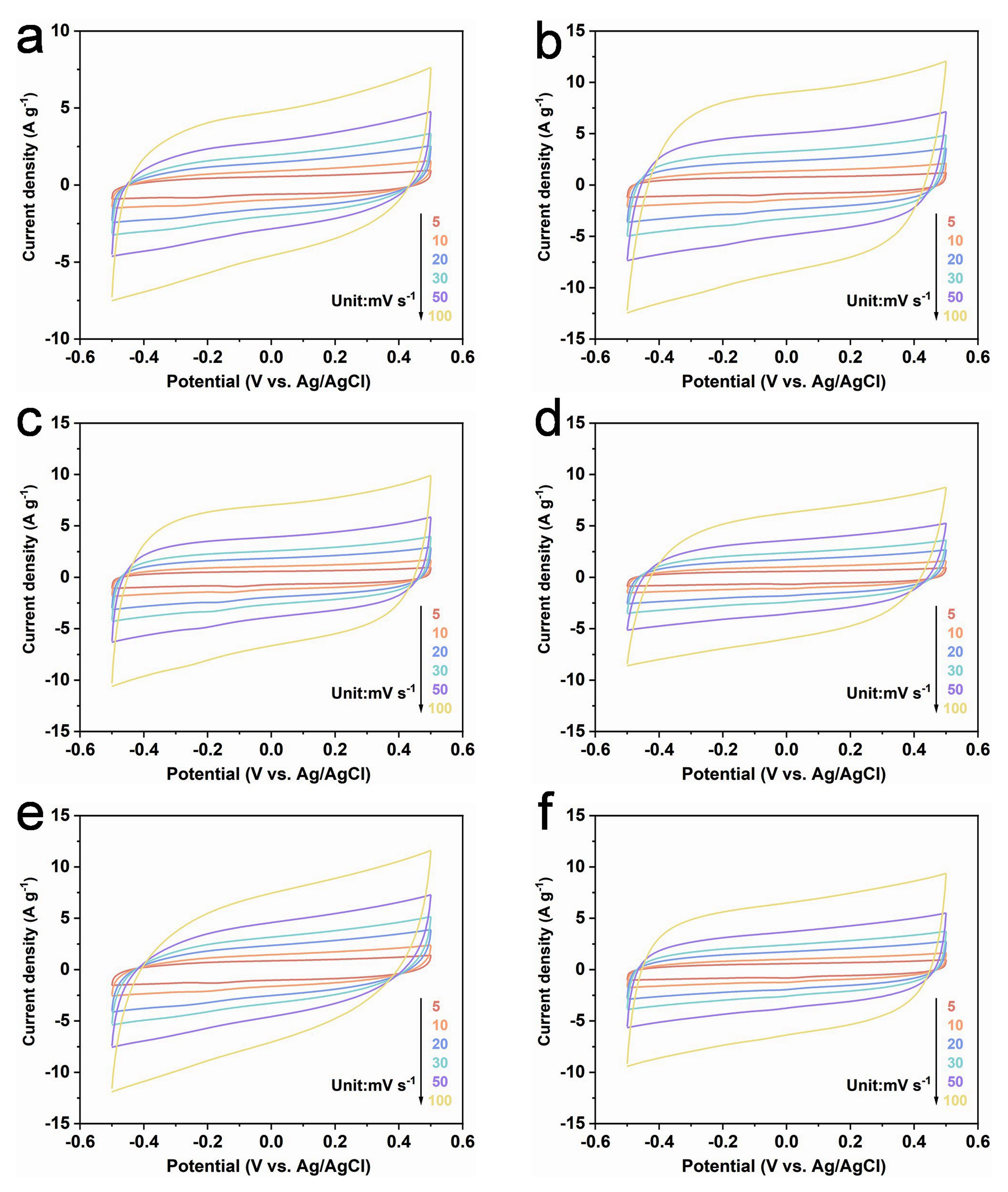


**Figure S23.** CV curves of (a) HMCSs (800, 5h), (b) NC(Zn)@HMCSs, (c) NC(Zn, Mn)@HMCSs, (d) NC(Zn, Co)@HMCSs, (e) NC(Zn, Ni)@HMCSs, (f) NC(Zn, Cu)@HMCSs at different scan rates.


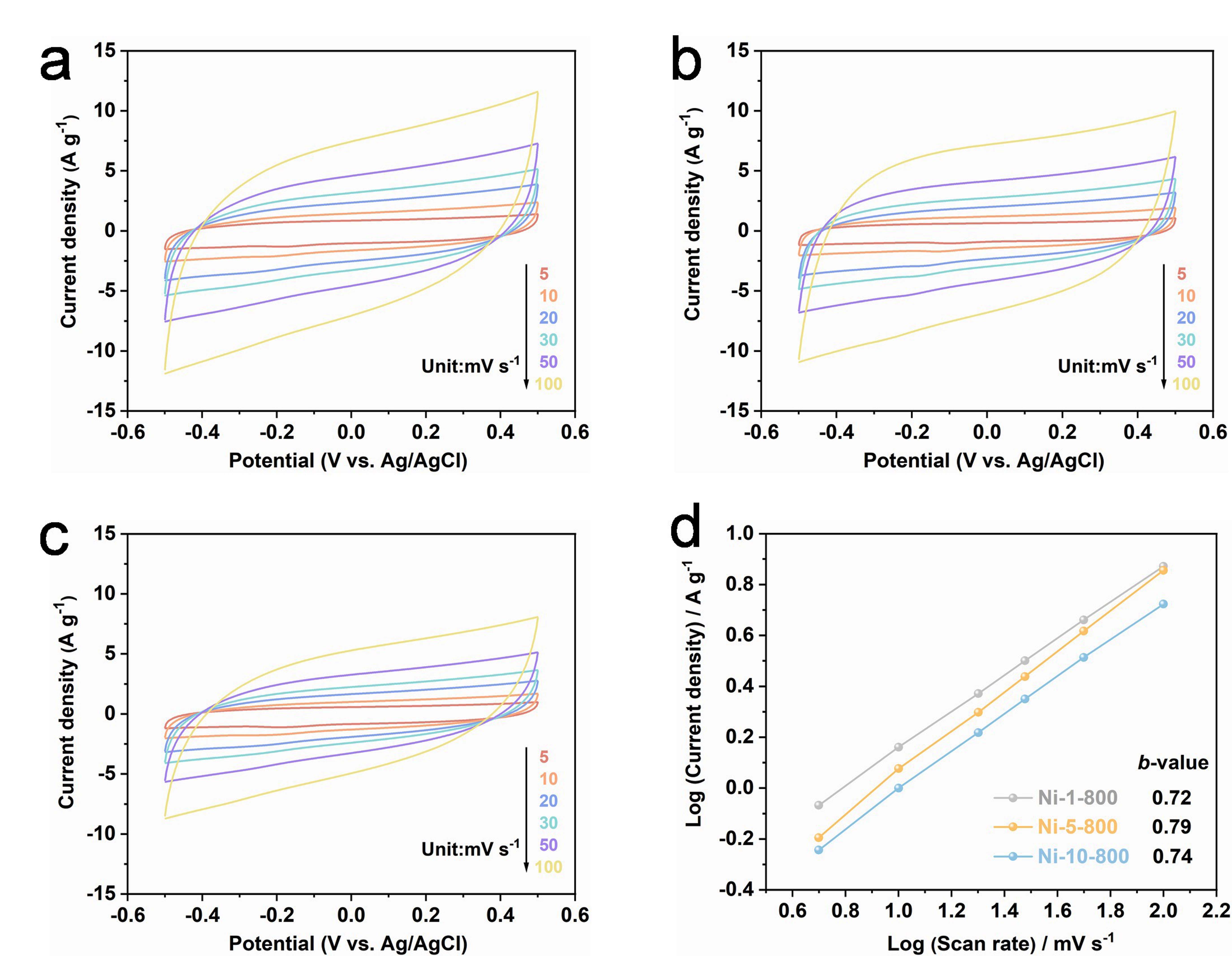


**Figure S24.** CV curves of (a) Ni-1-800, (b) Ni-5-800, (c) Ni-10-800 at different scan rates. (d) Log (current density) vs. Log (scan rate) charts of Ni-1-800, Ni-5-800, and Ni-10-800 electrodes in the charge process.


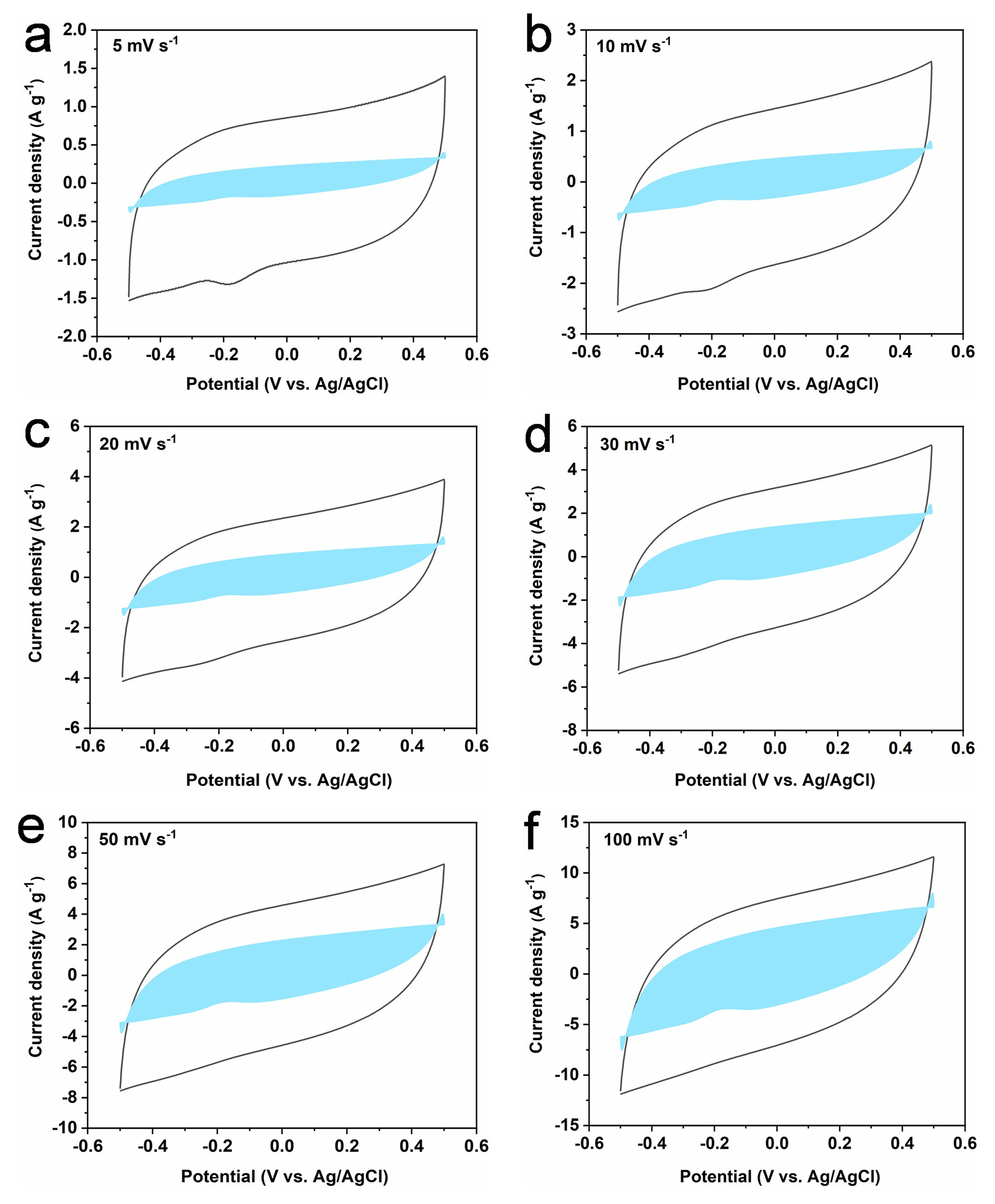


**Figure S25.** Decoupling of the diffusion-controlled (white) and capacitive (blue) contributions for Ni-1-800 electrode at different scan rates.


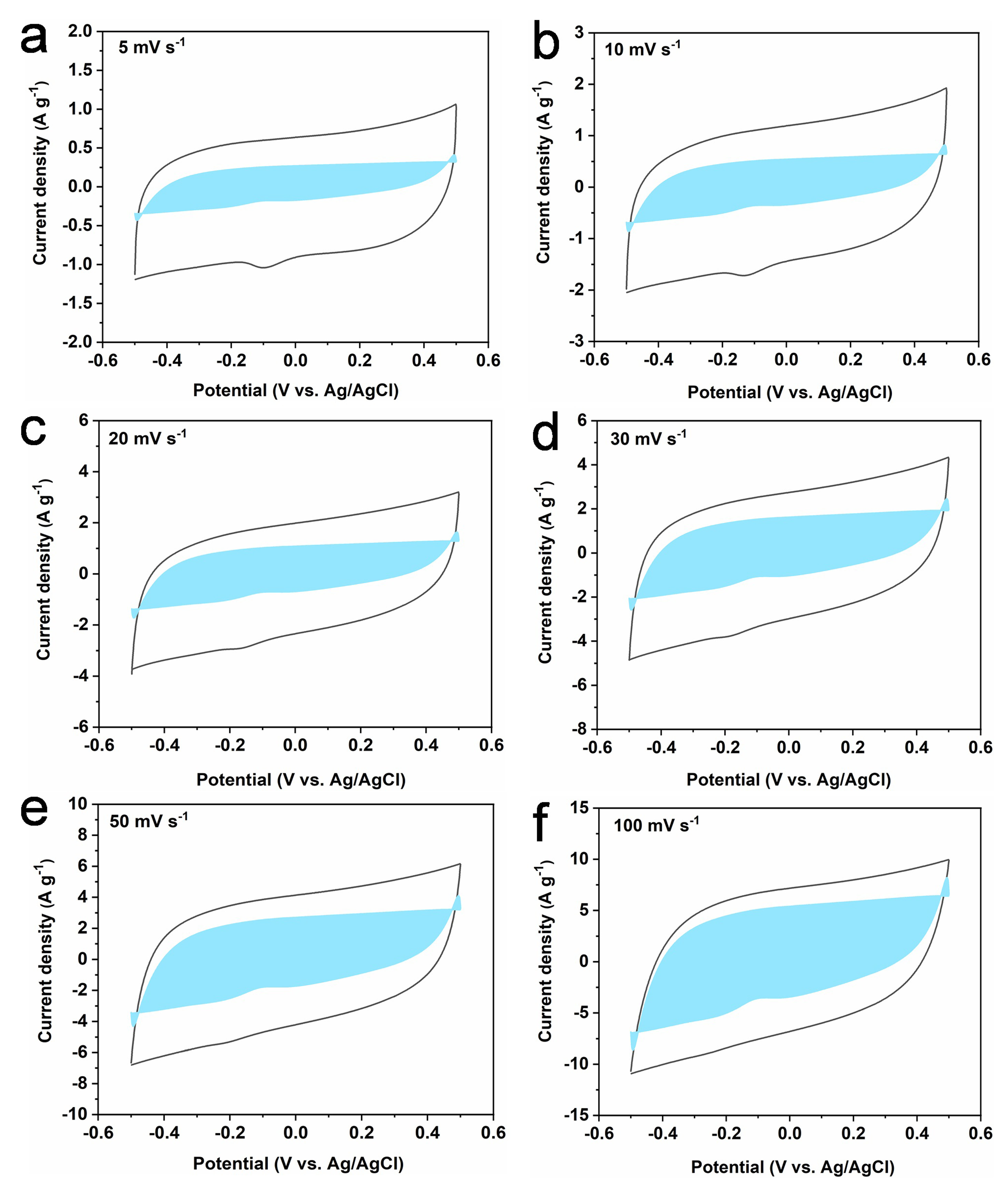


**Figure S26.** Decoupling of the diffusion-controlled (white) and capacitive (blue) contributions for Ni-5-800 electrode at different scan rates.


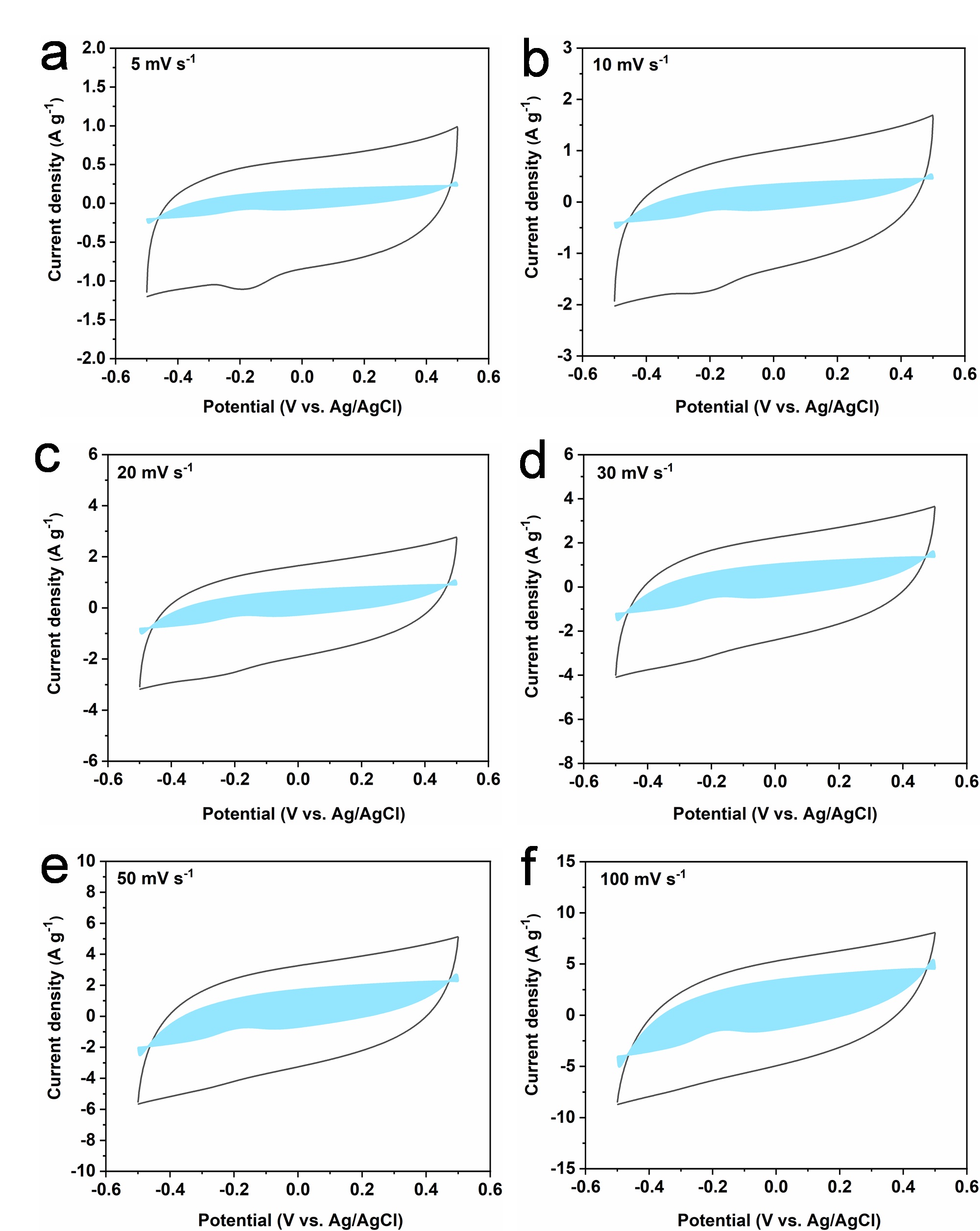


**Figure S27.** Decoupling of the diffusion-controlled (white) and capacitive (blue) contributions for Ni-10-800 electrode at different scan rates.


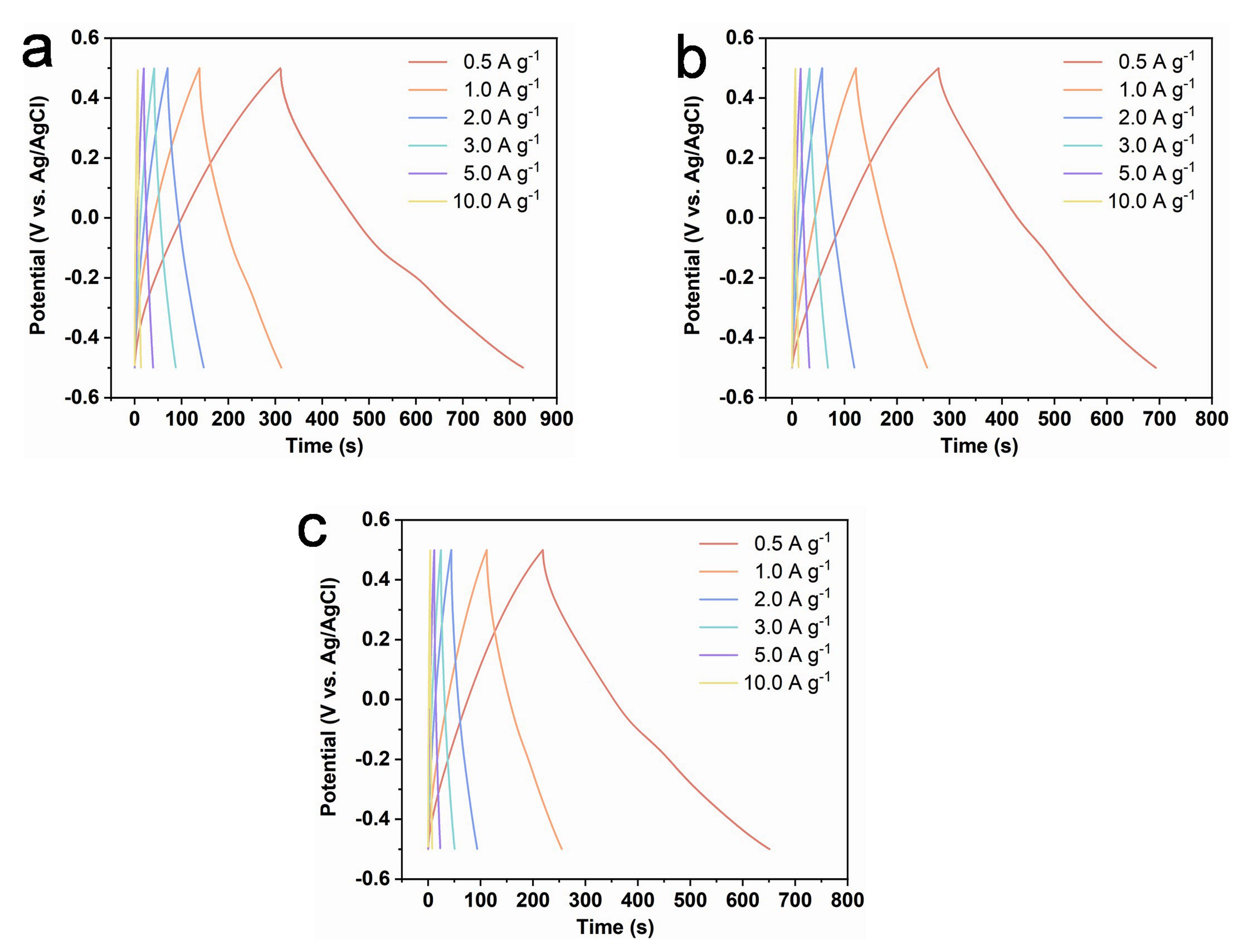


**Figure S28.** GCD curves of (a) Ni-1-800, (b) Ni-5-800, (c) Ni-10-800 at different current densities.


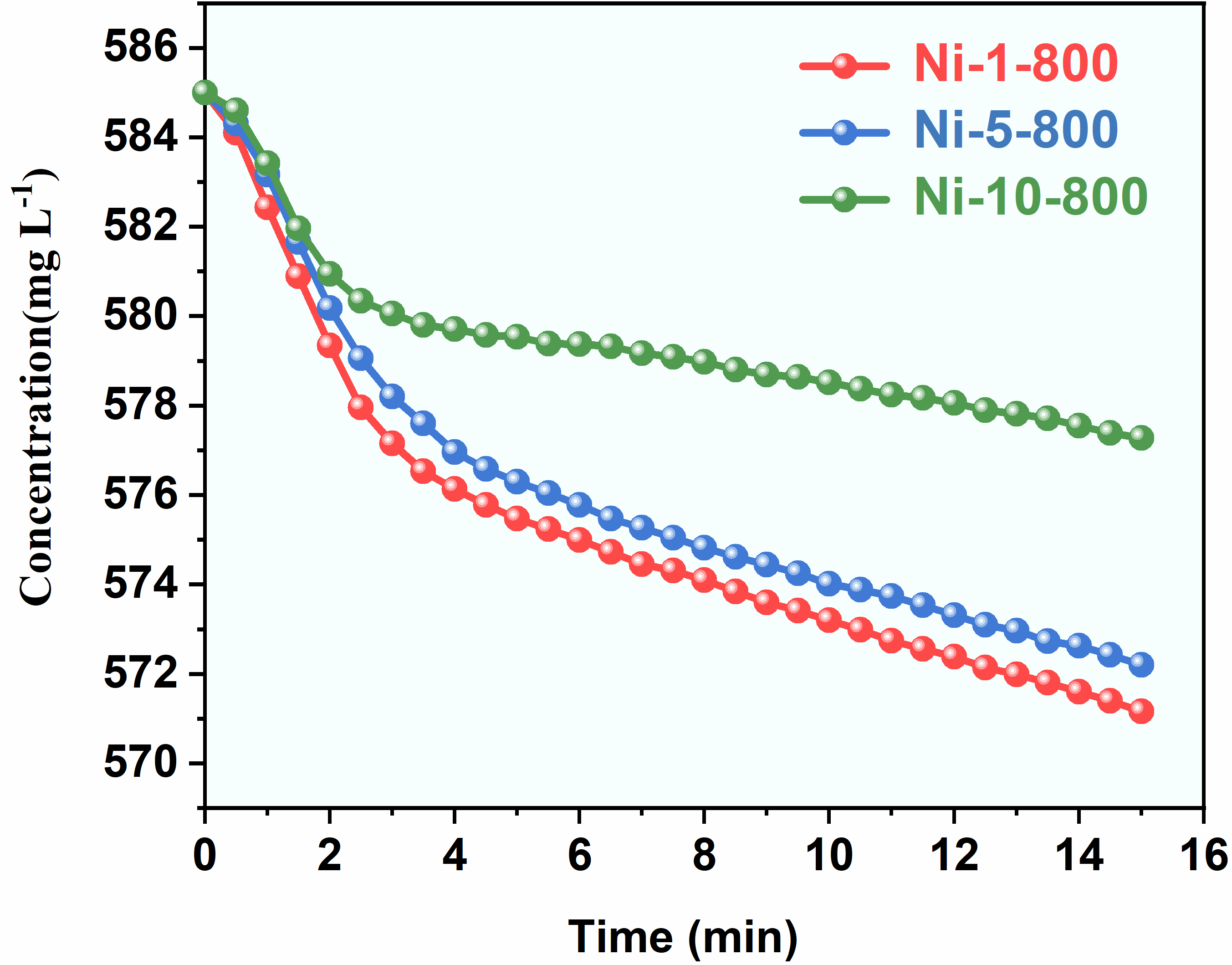


**Figure S29.** Concentration-time plots of Ni-1-800, Ni-5-800, and Ni-10-800 in 10.0 mM NaCl.


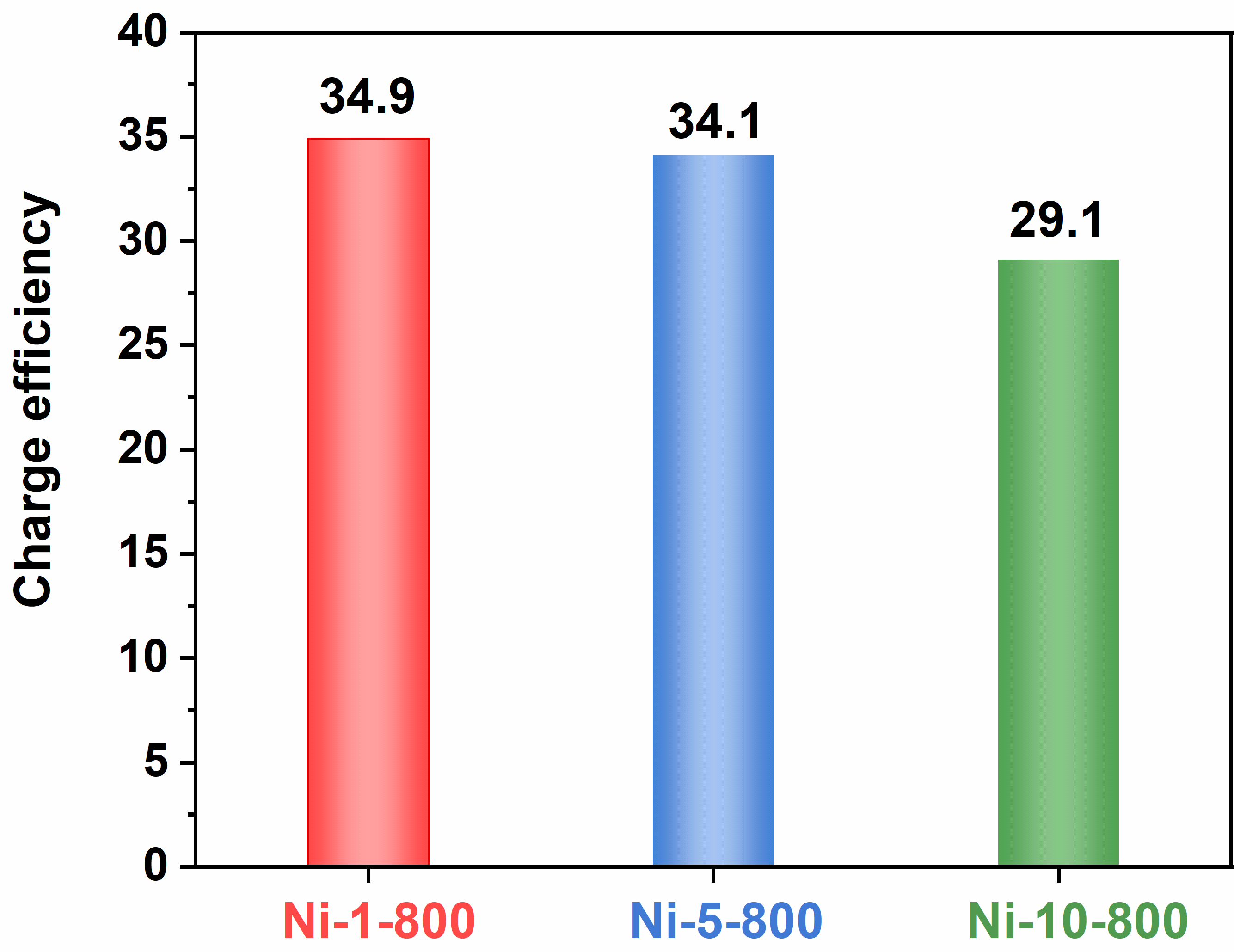


**Figure S30.** The calculated charge efficiencies (%) through current response of Ni-1-800, Ni-5-800, and Ni-10-800 electrodes.


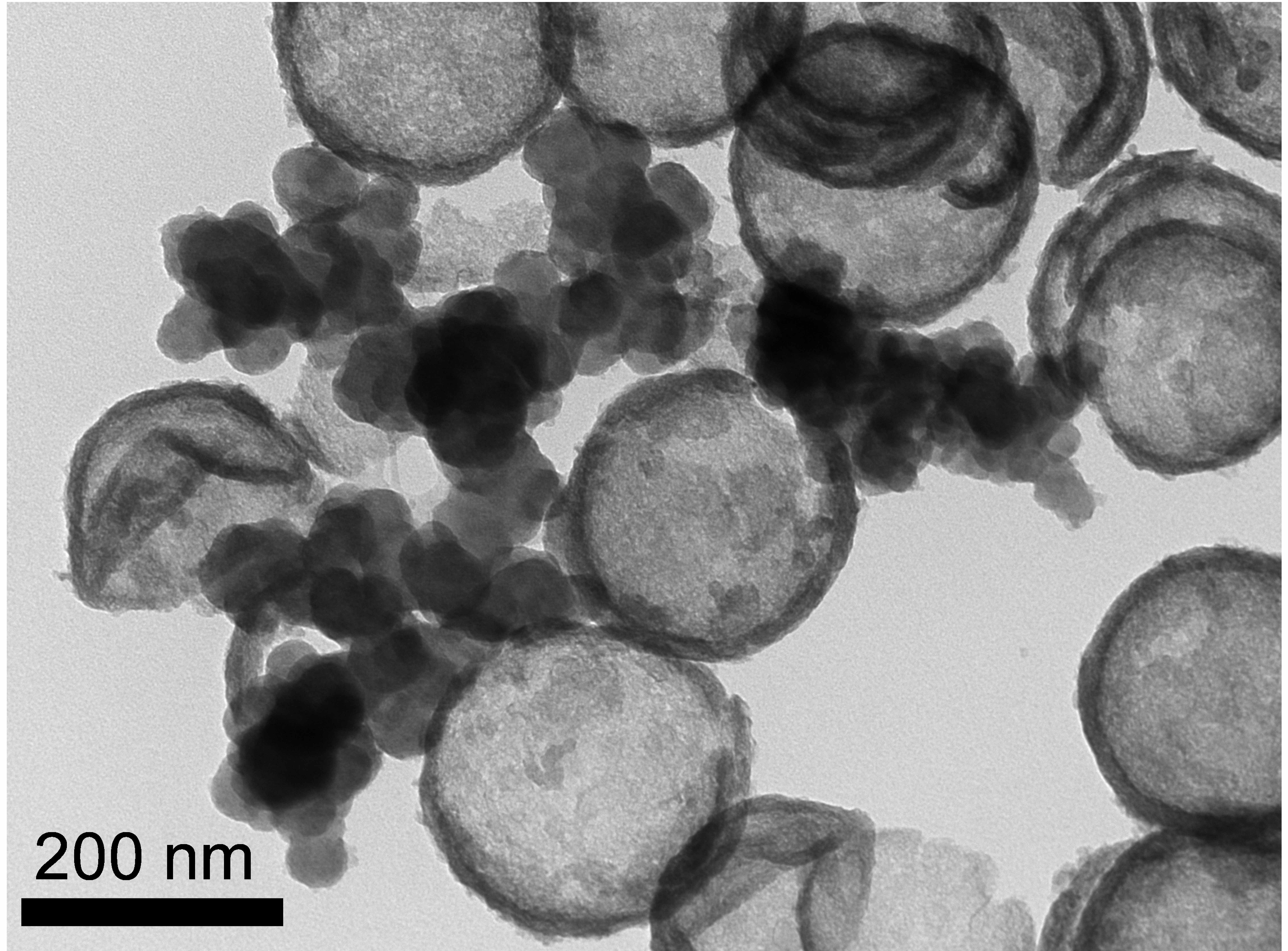


**Figure S31.** TEM image of Ni-1-800 after 20 CDI cycles.


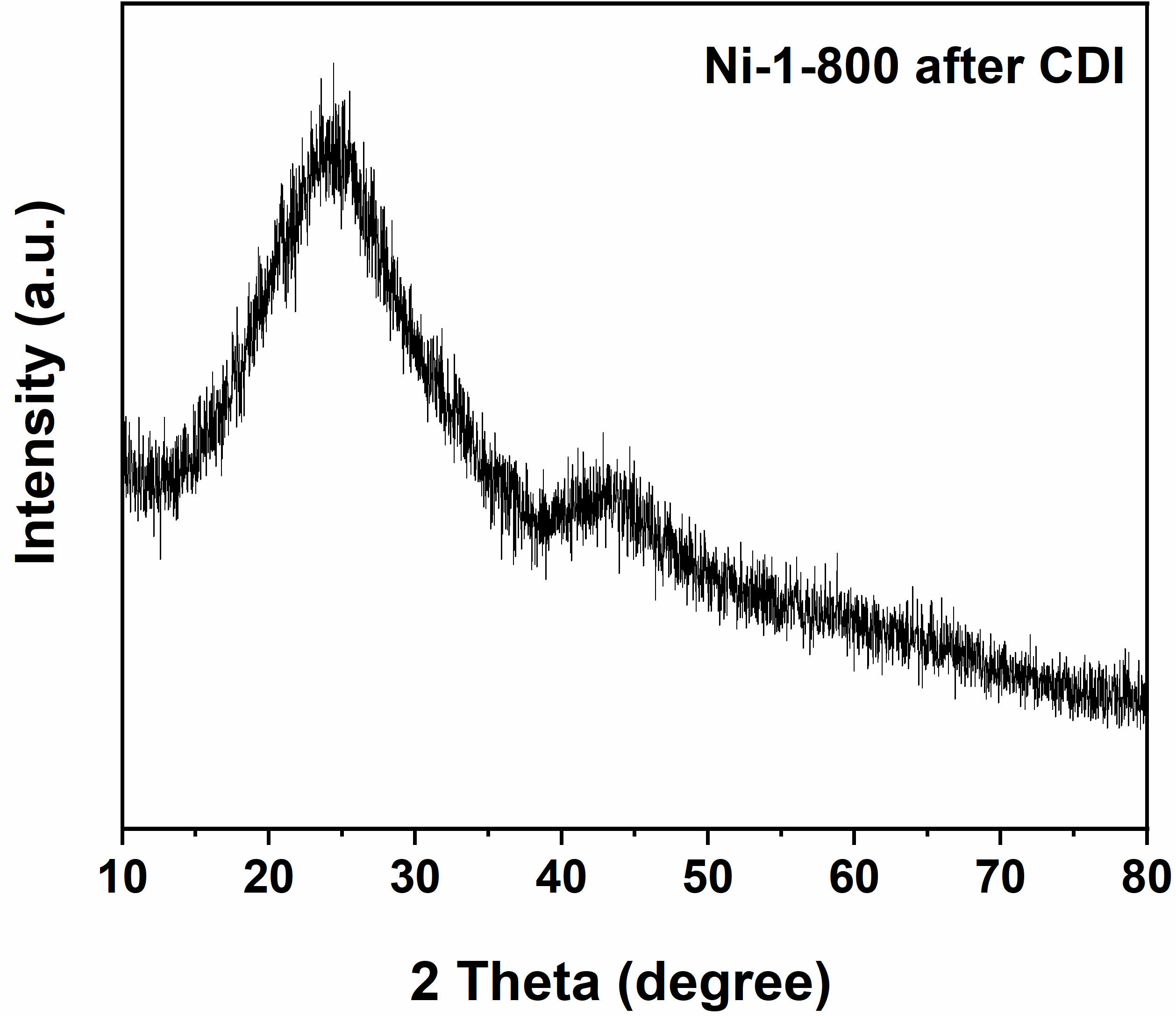


**Figure S32.** XRD pattern of Ni-1-800 after 20 CDI cycles.


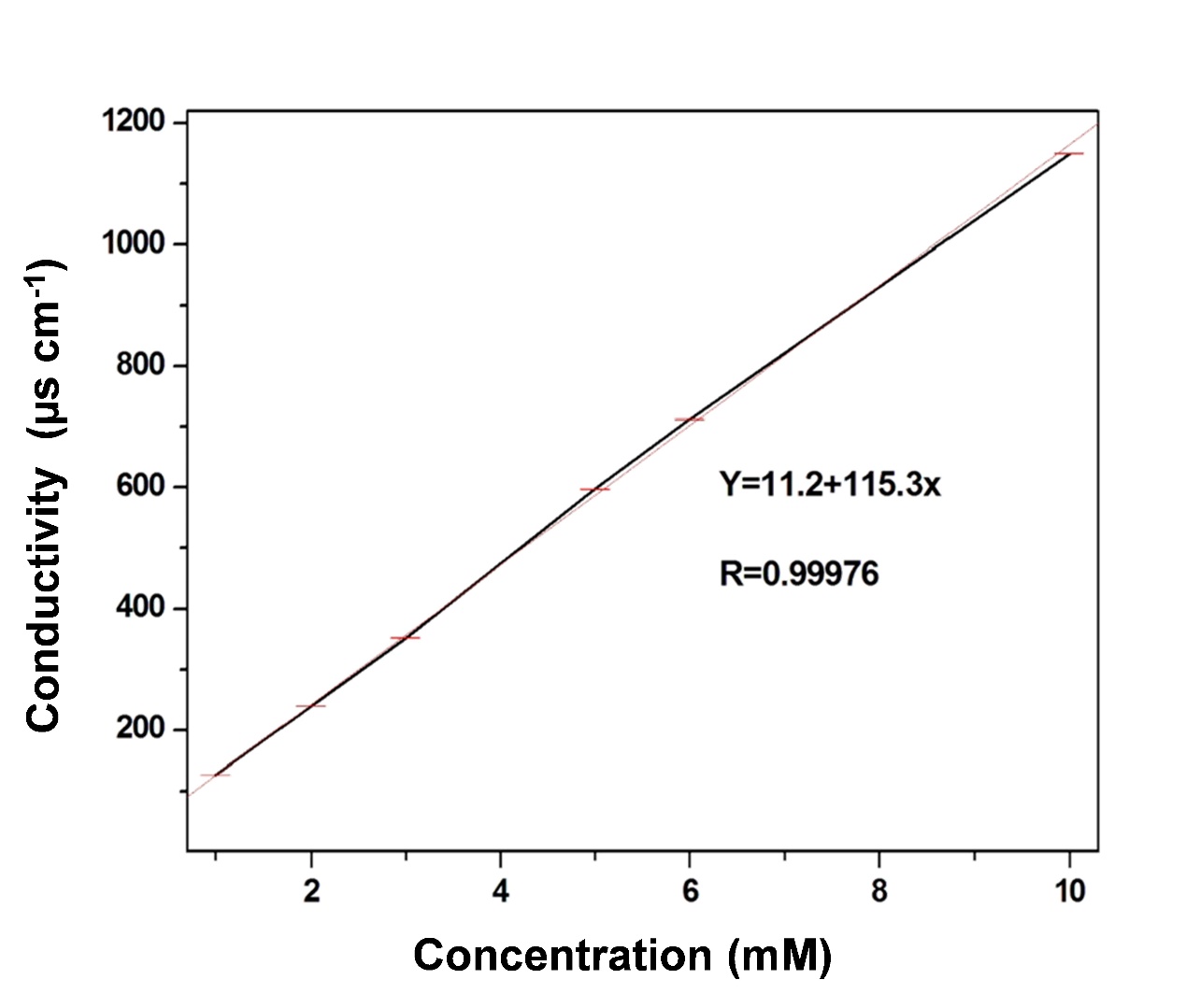


**Figure S33.** The relationship curve between concentration of NaCl solution and conductivity.

**Table S1.** Specific surface areas, pore volumes and mean pore diameters of hybrid materials.

| Sample | BET Surface Area (m^2^ g^-1^) | Pore Volume  (cm^3^ g^-1^) | Pore Size  (nm) |
| --- | --- | --- | --- |
| SiO_2_@SiO_2_+PDA | 192.556 | 0.121 | 3.536 |
| ZIF(Zn)/SiO_2_@SiO_2_+PDA | 219.762 | 0.136 | 3.355 |
| ZIF(Zn, Mn)/SiO_2_@SiO_2_+PDA | 210.634 | 0.138 | 3.584 |
| ZIF(Zn, Co)/SiO_2_@SiO_2_+PDA | 94.218 | 0.104 | 5.082 |
| ZIF(Zn, Ni)/SiO_2_@SiO_2_+PDA | 218.888 | 0.123 | 3.367 |
| ZIF(Zn, Cu)/SiO_2_@SiO_2_+PDA | 268.632 | 0.083 | 2.826 |
| HMCSs (800, 2h) | 1282.746 | 1.309 | 3.983 |
| HMCSs (800, 5h) | 1458.050 | 1.661 | 4.402 |
| ZIF(Zn)@HMCSs | 1219.594 | 0.915 | 3.782 |
| ZIF(Zn, Mn)@HMCSs | 1086.863 | 0.830 | 3.837 |
| ZIF(Zn, Co)@HMCSs | 1168.101 | 0.811 | 3.645 |
| ZIF(Zn, Ni)@HMCSs (Ni-1) | 1123.553 | 0.878 | 3.928 |
| Ni-5 | 1071.826 | 0.824 | 3.890 |
| Ni-10 | 1033.559 | 0.677 | 3.554 |
| ZIF(Zn, Cu)@HMCSs | 1096.625 | 0.913 | 4.093 |
| NC(Zn) | 557.355 | 0.524 | 5.266 |
| NC(Zn)/HMCSs | 1388.405 | 1.377 | 4.054 |
| NC(Zn)@HMCSs | 844.265 | 0.962 | 4.448 |
| NC(Zn, Ni)@HMCSs  (Ni-1-800) | 808.853 | 0.896 | 4.540 |
| Ni-5-800 | 765.314 | 0.889 | 4.786 |
| Ni-10-800 | 664.131 | 0.883 | 5.304 |

**Table S2.** The specific capacitance values (F g^-1^) with respect to scan rate calculated from respective CV curve.

| Scan rate  (mV s^-1^)  Samples | 5 | 10 | 20 | 30 | 50 | 100 |
| --- | --- | --- | --- | --- | --- | --- |
| HMCSs (800, 5h) | 108.4 | 87.5 | 69.9 | 61.7 | 52.9 | 43.1 |
| NC(Zn)@HMCSs | 154.6 | 132.6 | 112.8 | 103.0 | 91.6 | 76.6 |
| NC(Zn, Mn)@HMCSs | 124.1 | 107.8 | 91.5 | 83.3 | 74.2 | 63.0 |
| NC(Zn, Co)@HMCSs | 112.9 | 95.9 | 80.6 | 73.0 | 64.1 | 52.4 |
| NC(Zn, Ni)@HMCSs  (Ni-1-800) | 174.5 | 140.6 | 110.6 | 96.1 | 80.4 | 61.9 |
| Ni-5-800 | 144.3 | 121.6 | 98.8 | 87.3 | 75.0 | 60.0 |
| Ni-10-800 | 129.5 | 103.4 | 79.5 | 68.2 | 56.4 | 42.5 |
| NC(Zn, Cu)@HMCSs | 121.9 | 101.9 | 85.6 | 77.8 | 69.3 | 59.0 |
| NC (Zn) | 96.2 | 74.8 | 52.2 | 42.4 | 32.8 | 22.7 |
| HMCSs (800, 2h) | 95.2 | 79.6 | 63.1 | 55.3 | 46.6 | 36.2 |
| NC(Zn)/HMCSs | 131.5 | 118.1 | 104.7 | 97.9 | 89.8 | 78.9 |
| NC(Zn, Mn)/HMCSs | 92.0 | 79.9 | 69.1 | 63.6 | 56.9 | 47.8 |
| NC(Zn, Co)/HMCSs | 93.2 | 77.3 | 61.5 | 54.2 | 46.2 | 36.5 |
| NC(Zn, Ni)/HMCSs | 101.4 | 89.1 | 77.2 | 70.8 | 62.3 | 49.8 |
| NC(Zn, Cu)/HMCSs | 93.3 | 83.1 | 70.9 | 64.4 | 56.1 | 43.1 |

**Table S3.** The specific capacitance values (F g^-1^) with respect to current density calculated from respective GCD curve.

| Current density  (A g^-1^)  Samples | 0.5 | 1 | 2 | 3 | 5 | 10 |
| --- | --- | --- | --- | --- | --- | --- |
| HMCSs (800, 5h) | 150.6 | 113.6 | 80.2 | 64.8 | 51.5 | 38.2 |
| NC(Zn)@HMCSs | 199.9 | 166.4 | 134.2 | 117.9 | 99.5 | 82.3 |
| NC(Zn, Mn)@HMCSs | 169.2 | 123.6 | 101.8 | 90.3 | 76.5 | 62.9 |
| NC(Zn, Co)@HMCSs | 134.1 | 110.5 | 86.8 | 75.3 | 62.4 | 47.2 |
| NC(Zn, Ni)@HMCSs  (Ni-1-800) | 259.1 | 174.6 | 154.6 | 138.6 | 101.3 | 67.4 |
| Ni-5-800 | 206.7 | 136.1 | 122.8 | 105.3 | 83.1 | 61.2 |
| Ni-10-800 | 196.8 | 123.2 | 99.6 | 78.6 | 57.8 | 37.4 |
| NC(Zn, Cu)@HMCSs | 160.7 | 139.1 | 95.8 | 83.7 | 71.1 | 57.9 |
| NC (Zn) | 147.3 | 95.3 | 57.2 | 39.9 | 24.5 | 12.3 |
| HMCSs (800, 2h) | 135.9 | 95.8 | 67.2 | 54.9 | 42.5 | 28.3 |
| NC(Zn)/HMCSs | 169.2 | 138.6 | 116.8 | 106.8 | 94.5 | 81.1 |
| NC(Zn, Mn)/HMCSs | 111.2 | 88.8 | 72.4 | 65.1 | 55.6 | 43.2 |
| NC(Zn, Co)/HMCSs | 138.7 | 95.3 | 68.2 | 55.2 | 43.5 | 31.6 |
| NC(Zn, Ni)/HMCSs | 115.7 | 97.1 | 80.4 | 72.3 | 61.5 | 45.2 |
| NC(Zn, Cu)/HMCSs | 140.6 | 109.7 | 85.6 | 71.7 | 56.5 | 39.8 |

**Table S4.** Comparison of material adsorption capacity reported in the literature.

| Electrode material | Operating voltage (V) | Initial NaCl concentration (mg L^-1^) | SAC  (mg g^-1^) | Ref. |
| --- | --- | --- | --- | --- |
| N-HMCSs | 1.6 | 500 | 16.6 | [1] |
| NCPC-900 | 1.6 | 500 | 17.2 | [2] |
| CHS 1 | 1.6 | 250 | 18.8 | [3] |
| HCB | 1.4 | 450 | 21.8 | [4] |
| NPHCS | 1.4 | 500 | 12.95 | [5] |
| HAC-1 | 1.2 | 500 | 29.5 | [6] |
| TiS_2_@HCSs | 1.2 | 100 | 18.0 | [7] |
| N-CHS | 1.2 | 250 | 13.38 | [8] |
| NSHPC | 1.2 | 500 | 18.71 | [9] |
| A-HCMs | 1.0 | 400 | 14.64 | [10] |
| NC-800 | 1.2 | 58 | 8.52 | [11] |
| Ni_3_Si_2_O_5_(OH)_4_/C | 1.2 | 584 | 28.7 | [12] |
| Ag/ZCs | 1.2 | 500 | 29.18 | [13] |
| HMCSs (800, 2h) | 1.2 | 584 | 19.1 | This work |
| HMCSs (800, 5h) | 1.2 | 584 | 23.9 | This work |
| NC(Zn)/HMCSs | 1.2 | 584 | 28.9 | This work |
| NC(Zn)@HMCSs | 1.2 | 584 | 32.1 | This work |
| Ni-1-800 | 1.2 | 584 | 37.8 | This work |
| Ni-5-800 | 1.2 | 584 | 34.6 | This work |
| Ni-10-800 | 1.2 | 584 | 29.2 | This work |

**SAC:** **salt adsorption capacity**

**References**

[1] Y. Li, J. Qi, J. Li, J. Shen, Y. Liu, X. Sun, J. Shen, W. Han, L. Wang, *ACS Sustainable Chemistry & Engineering* **2017**, *5* (8), 6635, https://doi.org/10.1021/acssuschemeng.7b00884.

[2] Y. Li, Y. Liu, J. Shen, J. Qi, J. Li, X. Sun, J. Shen, W. Han, L. Wang, *Desalination* **2018**, *430*, 45, https://doi.org/10.1016/j.desal.2017.12.040.

[3] Z. Y. Leong, H. Y. Yang, *RSC Advances* **2016**, *6* (58), 53542, https://doi.org/10.1039/c6ra06489b.

[4] X. Zang, Z. Fu, D. Wang, Z. Yuan, N. Shi, Z. Yang, Y.-M. Yan, *Journal of Materials Chemistry A* **2022**, *10* (18), 9988, https://doi.org/10.1039/d2ta00611a.

[5] S. Zhao, T. Yan, H. Wang, G. Chen, L. Huang, J. Zhang, L. Shi, D. Zhang, *Applied Surface Science* **2016**, *369*, 460, https://doi.org/10.1016/j.apsusc.2016.02.085.

[6] M. Kim, X. Xu, R. Xin, J. Earnshaw, A. Ashok, J. Kim, T. Park, A. K. Nanjundan, W. A. El-Said, J. W. Yi, J. Na, Y. Yamauchi, *ACS Appl Mater Interfaces* **2021**, *13*(44), 52034, https://doi.org/10.1021/acsami.1c09107.

[7] M. Ezzati, F. Hekmat, S. Shahrokhian, H. E. Unalan, *Desalination* **2022**, *533*, 115766, https://doi.org/10.1016/j.desal.2022.115766.

[8] M. Ding, F.-H. Du, B. Liu, Z. Y. Leong, L. Guo, F. Chen, A. Baji, H. Y. Yang, *FlatChem* **2018**, *7*, 10, https://doi.org/10.1016/j.flatc.2018.01.002.

[9] Y. Huang, J. Yang, L. Hu, D. Xia, Q. Zhang, Y. Liao, H. Li, W. Yang, C. He, D. Shu, *Environmental Science: Nano* **2019**, *6* (5), 1430, https://doi.org/10.1039/c9en00028c.

[10] P. Wang, W. Ma, S. Xue, L. Wang, Y. Chen, Y. Wang, *Separation and Purification Technology* **2021**, *276*, 119336, https://doi.org/10.1016/j.seppur.2021.119336.

[11] N. L. Liu, S. Dutta, R. R. Salunkhe, T. Ahamad, S. M. Alshehri, Y. Yamauchi, C. H. Hou, K. C. Wu, *Sci Rep* **2016**, *6*, 28847, https://doi.org/10.1038/srep28847.

[12] Y. Tang, S. Zheng, S. Cao, F. Yang, X. Guo, S. Zhang, H. Xue, H. Pang, *J Colloid Interface Sci* **2022**, *626*, 1062, https://doi.org/10.1016/j.jcis.2022.07.034.

[13] H. Zhang, W. Zhang, J. Shen, Y. Li, X. Yan, J. Qi, X. Sun, J. Shen, W. Han, L. Wang, J. Li, *Desalination* **2020**, *473*, 114173 https://doi.org/10.1016/j.desal.2019.114173.
